# Physiological Response of *Corynebacterium glutamicum* to Indole

**DOI:** 10.3390/microorganisms8121945

**Published:** 2020-12-08

**Authors:** Tatjana Walter, Kareen H. Veldmann, Susanne Götker, Tobias Busche, Christian Rückert, Arman Beyraghdar Kashkooli, Jannik Paulus, Katarina Cankar, Volker F. Wendisch

**Affiliations:** 1 Genetics of Prokaryotes, Faculty of Biology and CeBiTec, Bielefeld University, 33615 Bielefeld, Germany; t.walter@uni-bielefeld.de (T.W.); veldmann.kareen@gmx.de (K.H.V.); sgoetker@cebitec.uni-bielefeld.de (S.G.); 2 Center for Biotechnology, Bielefeld University, 33615 Bielefeld, Germany; tbusche@cebitec.uni-bielefeld.de (T.B.); cruecker@CeBiTec.Uni-Bielefeld.DE (C.R.); 3 BU Bioscience, Wageningen University & Research, 6700AA Wageningen, The Netherlands; a.beyraghdar@yahoo.com (A.B.K.); katarina.cankar@wur.nl (K.C.); 4 Organic and Bioorganic Chemistry, Department of Chemistry, Bielefeld University, 33615 Bielefeld, Germany; j.paulus@uni-bielefeld.de

**Keywords:** *Corynebacterium glutamicum*, amino acids, indole, adaptive laboratory evolution, iron homeostasis, oxidative stress, aromatic compound catabolism

## Abstract

The aromatic heterocyclic compound indole is widely spread in nature. Due to its floral odor indole finds application in dairy, flavor, and fragrance products. Indole is an inter- and intracellular signaling molecule influencing cell division, sporulation, or virulence in some bacteria that synthesize it from tryptophan by tryptophanase. *Corynebacterium glutamicum* that is used for the industrial production of amino acids including tryptophan lacks tryptophanase. To test if indole is metabolized by *C. glutamicum* or has a regulatory role, the physiological response to indole by this bacterium was studied. As shown by RNAseq analysis, indole, which inhibited growth at low concentrations, increased expression of genes involved in the metabolism of iron, copper, and aromatic compounds. In part, this may be due to iron reduction as indole was shown to reduce Fe^3+^ to Fe^2+^ in the culture medium. Mutants with improved tolerance to indole were selected by adaptive laboratory evolution. Among the mutations identified by genome sequencing, mutations in three transcriptional regulator genes were demonstrated to be causal for increased indole tolerance. These code for the regulator of iron homeostasis DtxR, the regulator of oxidative stress response RosR, and the hitherto uncharacterized Cg3388. Gel mobility shift analysis revealed that Cg3388 binds to the intergenic region between its own gene and the *iolT2-rhcM2D2* operon encoding inositol uptake system IolT2, maleylacetate reductase, and catechol 1,2-dioxygenase. Increased RNA levels of *rhcM2* in a *cg3388* deletion strain indicated that Cg3388 acts as repressor. Indole, hydroquinone, and 1,2,4-trihydroxybenzene may function as inducers of the *iolT2-rhcM2D2* operon in vivo as they interfered with DNA binding of Cg3388 at physiological concentrations in vitro. Cg3388 was named IhtR.

## 1. Introduction

Indole is a bioactive aromatic compound and used as flavor and fragrance in the cosmetics (e.g., perfume) and food (e.g., dairy products) industries because of its floral odor which is typical for jasmine [1]. The hormone indole-3-acetic acid facilitates plant growth and finds application in the agricultural industry either directly or via plant growth promoting bacteria which were for example found in the rhizosphere from *Stevia rebaudiana* [2]. Indigo, the main coloring dye for denim, arises from oxidation of indole to indoxyl followed by oxidative dimerization [3].

In nature, the versatile widely distributed signaling molecule indole has significant roles in bacterial physiology, pathogenesis, animal behavior, and human diseases [4]. In bacteria, indole is known as intra- and intercellular signaling molecule that modulates diverse processes including plasmid stability, cell division, antibiotic tolerance, virulence, and spore formation [4,5]. Indole facilitates growth of plants, their root development or increased seedling growth, and functions in the response to herbivore attacks [4]. Animals cannot synthesize indole but can sense and modify indole. Indole is present in the gastrointestinal tract, the brain, or the blood of humans and might influence diseases such as cancers or bacterial infections [4,5]. In plants, indole-3-glycerol phosphate lyases (IGLs) cleave indole-3-glycerol to yield indole and d-glyceraldehyde-3-phosphate [6]. In bacteria, tryptophanases (TNAs) convert the amino acid l-tryptophan to indole, pyruvate, and ammonia [7].

Different physiological responses to extracellular addition or intracellular synthesis of indole have been described for indole-producing as well as for non-indole-producing bacteria affecting growth, biofilm formation, antibiotic resistance, acid and heat resistance, or indole persistence [5]. These effects may vary, e.g., biofilm formation is increased by indole in *E. coli* and *Pseudomonas putida*, but decreased in *Paenibacillus alvei*. Similarly, tolerance to antibiotics is decreased by indole in *Staphylococcus aureus* and *Vibrio anguillarum*, but increased in *Agrobacterium tumefaciens* and *Vibrio cholera* [5]. Thus, while indole often has profound effects on bacteria, it remains to be studied if these are detrimental or beneficial. Transport of indole across the bacterial cell membrane has been best studied in *E. coli*, where Mtr [8,9] and Mtr independent uptake of indole by diffusion [10] have been found. Export of indole may involve AcrEF [11].

Aerobic degradation of indole proceeds via the two key intermediates: isatin and anthranilate [12,13]. Several aromatic oxygenases, such as phenol hydroxylase and cytochrome P450 hydroxylase, can oxidize indole (at C2 or C3 position) to yield indoxyls, which are further oxidized to indigoids [12,13]. The first potential indole-specific hydrolases were recently identified in *Acinetobacter* [14] and *Cupriavidus* [15]. A gene cluster responsible for indole upstream metabolism to produce anthranilate was identified in both bacteria. Indole is degraded to anthranilate by the indole oxygenase with oxygenase and flavin reductase subunits, followed by a short-chain dehydrogenase and a cofactor independent oxygenase [14]. Indole degradation under anaerobic conditions occurs with tryptophan and isatin as key intermediates. The main difference between both conditions is the hydroxylation at the C2 position leading to 2-oxiindole [12,13].

The knowledge about the genetic mechanism underlying the physiological response in bacteria is limited. Most studies were focused on the visible changes in the physiological response on indole and only a few about the transcriptomic response [16,17]. *C. glutamicum*, which is used for the industrial production of amino acids including the aromatic amino acid l-tryptophan, does not synthesize indole, but is able to utilize a number of other aromatic compounds for growth [18,19,20,21,22,23,24,25,26,27,28]. The degradation pathways mainly share the two intermediates catechol and protocatechuate (PCA), which via β-ketadipate enter the central carbon metabolism as succinyl-CoA and acetyl-CoA [18,29]. This so-called β-ketoadipate pathway is widely distributed among bacteria and serves as main degradation pathway in *C. glutamicum*. The degradation of resorcinol and 2,4-dihydroxybenzoate also occurs via this pathway, using 1,2,4-trihydroxybenzene as intermediate [23,24,30]. In contrast, naphthalene is converted via gentisate to fumarate and pyruvate [19,31]. Transport systems [32,33,34,35], transcriptional regulators [36,37,38,39,40,41], and production of aromatic compounds [42,43,44,45,46,47,48] have been described for *C. glutamicum*. In order to test if indole is metabolized by *C. glutamicum* or exerts a regulatory role as a putative signaling molecule, we determined the physiological and transcriptomic response of *C. glutamicum* to indole.

## 2. Materials and Methods

### 2.1. Bacterial Strains and Molecular Genetic Techniques

All bacterial strains used are listed in Table 1. *E. coli* DH5α [49] was used for plasmid construction. *C. glutamicum* WT and C1* were used for investigation of indole response. Standard molecular genetic techniques were performed as described in [50]. Competent *E. coli* DH5α [49] was performed with the RbCl method and transformed by heat shock [50]. Transformation of *C. glutamicum* was performed by electroporation [51].

Chromosomal gene deletions, replacements, and base exchanges in *C. glutamicum* were performed by two-step homologous recombination [51] using the suicide vector pK19*mobsacB* [58]. The genomic regions flanking the respective gene for homologous recombination were amplified from *C. glutamicum* as described elsewhere [50] using the respective primer pairs (Supplementary Data Table S1). The purified PCR products were assembled and simultaneously cloned into restricted pK19*mobsacB* by Gibson Assembly resulting in the plasmids listed in Supplementary Data Table S2. For construction of 19*mobsacB-*Δ*dtxR* and pK19*mobsacB-*Δ*rosR*, the strains Δ*dtxR* and Δ*rosR* were used as templates for fragment amplification by PCR. Transfer of the suicide vectors was carried out by transconjugation using *E. coli* S17 as donor strain [56]. For the first recombination event, integration of the vector in one of the targeted flanking regions was selected via kanamycin resistance. The resulting clones showed sucrose sensitivity due to levansucrase gene *sacB*. Suicide vector excision was selected by sucrose resistance. Gene deletions or replacements were verified by PCR and sequencing with respective primers (Supplementary Data Table S1). Overexpression of genes with artificial optimized ribosomal binding sites (RBS) [59] in *C. glutamicum* C1* was performed with the *C. glutamicum/E. coli* shuttle vector pVWEx1 or pEKEx3. Protein expression for purification was done with the overexpression vector pET-16b (Novagen, Merck Group, Darmstadt, Germany).

### 2.2. Culture Conditions

Precultures of *E. coli* and *C. glutamicum* were performed in lysogeny broth (LB) at 37 or 30 °C in baffled shake flasks on a rotary shaker (160 or 120 rpm). Cultures were inoculated freshly from LB agar plates. When necessary, kanamycin (25 µg m L^−1^) or spectinomycin (100 µg m L^−1^) were added to the medium. For induction of gene expression from vectors pVWEx1 or pEKEx3 1 mM isopropyl-β-d-1-thiogalactopyranoside (IPTG) was added to the medium. For growth or production experiments with *C. glutamicum*, precultures as described above were harvested (3200× *g*, 7 min), cells were washed with TN-buffer pH 6.3 (50 mM Tris-HCL, 50 mM NaCl) and inoculated to an optical density at 600 nm (OD_600_) of 1 in CGXII minimal medium [51] with 40 g L^−1^ glucose as sole carbon source if not otherwise noted. *C. glutamicum* was grown in 500 mL or 100 mL baffled shake flasks or in Micro-Flask microtiter plates (24-square deep-well polypropylene, 17 × 17 mm, depth 40 mm, Applikon Biotechnology, Delft, The Netherlands) at 30 °C and 120 or 220 rpm, followed by measuring OD_600_ using V-1200 spectrophotometer (VWR, Radnor, PA, USA) or microbioreactor system Biolector (m2p-labs; Aachen, Germany). For growth in the Biolector system, cultures were grown in LB-rich medium overnight and transferred to second preculture of CGXII minimal medium with 40 g L^−1^ glucose with addition of indole, if required until early exponential phase before inoculating to the main medium of CGXII minimal medium and 40 g L^−1^ glucose. Growth experiments in the Biolector system were carried out using 48-well flower plates (MTP-48-B; m2p-labs; Aachen, Germany) with a filling volume of 1 mL, at 30 °C, and 1200 rpm shaking frequency. Humidity was kept constant at 85%, and online biomass measurements of scattered light were monitored with backscatter gain of 30.

For mRNA isolation, *C. glutamicum* WT or C1* were cultivated in triplicates in CGXII minimal medium with 40 g L^−1^ glucose with addition of either ±2.5 or ±4 mM indole (Ind, dissolved in ethanol), ±3 mM indole-alanine TFA (Ind-Ala, dissolved in water), ±2.5 mM resorcinol (Res, dissolved in water), or ±2.5 mM myo-inositol (Ino, dissolved in water). Cultivation was performed in 100 mL baffled shake flasks at 220 rpm at 30 °C for all conditions. For the cultivation with ±2.5 mM indole, 500 mL baffled shake flaks were used. For transcription analysis by RNA sequencing, exponentially growing cells (OD_600_ of 4 for ±2.5 mM indole, OD_600_ of 6 for the other conditions) were collected to 50 mL falcons filled with ice and centrifuged at 4 °C for 10 min at 3.220× *g*. For transcription analysis by qRT-PCR, 2 mL of culture was shortly spin down in precooled tubes at 4 °C at 3.220× *g*. The resulting cell pellets were frozen in liquid nitrogen and stored at −80 °C until further use. Growth was monitored in independent parallel cultures.

For adaptive laboratory evolution, *C. glutamicum* WT was cultivated in triplicates using 100 mL baffled shaking flasks at 120 rpm and 30 °C in CGXII minimal medium with 40 g L^−1^ glucose with different concentrations of indole (dissolved in ethanol). The culture reaching the highest OD_600_ after 24 or 72 h was harvested and washed with TN buffer and used to inoculate three new cultures. The indole concentration in the medium was increased when growth OD_600_ values were observed in the previous culture. In total, 38 transfers were done. Evolved strains were stored as glycerol culture at −80 °C.

### 2.3. Sequencing of Transcriptomis and Genomic Data

#### 2.3.1. RNA Isolation, qRT-PCR, Preparation of cDNA Libraries for Sequencing and DeSeq Analysis

In order to isolate total RNA from *C. glutamicum* cells, bacterial cell pellets previously harvested and kept at −80 °C were thawed on ice and RNA was extracted individually for each cultivation condition using a NucleoSpin RNA isolation kit (Macherey-Nagel, Düren, Germany). Polymerase chain reactions with Taq polymerase (New England Biolabs, Frankfurt, Germany) were performed to detect if contaminating genomic DNA remained in the samples. RNA samples with genomic DNA contamination were treated with RNase-free DNase (Qiagen, Hilden, Germany). Total RNA concentrations were measured using a spectrophotometer (NanoDrop^®^, ND-1000; ThermoFisher Scientific, Schwerte, Germany). RNA quality was checked by Trinean Xpose (Gentbrugge, Belgium) and Agilent RNA Nano 6000 kit on Agilent 2100 Bioanalyzer (Agilent Technologies, Böblingen, Germany). The extracted RNA samples were either pooled (treatment with indole) or separately treated (treatment with Ind-Ala). Ribo-Zero rRNA Removal Kit (Bacteria) from Illumina (San Diego, CA, USA) was used to remove the ribosomal RNA molecules from the isolated total RNA. Removal of rRNA was checked by Agilent RNA Pico 6000 kit on an Agilent 2100 Bioanalyzer (Agilent Technologies). RNA was free of detectable rRNA. Preparation of cDNA libraries were performed according to the manufacturer’s instructions of the TruSeq stranded mRNA Kit (Illumina). Subsequently, each cDNA library was sequenced on a HiSeq1500 (2 × 70 nt PE rapid v2) Sequencer system (Illumina). The software Bowtie2 [60] was used for mapping to the respective genomes (BA000036 for WT, NZ_CP017995.1 for C1*). In order to perform differential gene expression analysis, DEseq2 for separately treated samples and DEseq for pooled samples [61,62] were used as a part of the software ReadXplorer(2) [63,64]. Statistically significant expression changes (adjusted *p*-value ≤ 0.01) with log2 fold change >1.5 or <−1.5 were used. The transcriptomic data are available via NCBI GEO accession identifiers GSE159887 and GSE159888.

All qRT-PCRs were performed according to the manufacturer’s instruction using the SensiFASTTM SYBR^®^ No-ROX One-Step Kit (Meridian bioscience, Ohio, USA) and the CFX96 cycler system (Bio-Rad). The temperature profile was (1) 45 °C for 10 min (reverse transcription); (2) 95 °C for 2 min; (3) 40 cycles of 95 °C for 5 s, 55 °C for 10 s, and 70 °C for 5 s; (4) melt curve analysis with measures between 65 and 95 °C. The log2 fold change of the negative ΔΔCq (reference Cq–sample Cq) value, using the reference gene *parA* (cg3427), was used in calculations [65,66]. For each sample, three independent qRT-PCR experiments were performed.

#### 2.3.2. gDNA Isolation, Library Preparation and Sequencing

For isolation of genomic DNA (gDNA), *C. glutamicum* WT and evolved strains were cultivated in triplicates in LB medium 100 mL baffled shake flasks at 120 rpm and 30 °C overnight and the complete culture harvested. Genomic DNA was isolated using the NucleoSpin Microbial DNA kit for DNA, RNA, and protein purification (Macherey-Nagel) according to the manufacturer. Quality of isolated gDNA was analyzed using a spectrophotometer (NanoDrop^®^, ND-1000). The complete digestion of the RNA was verified by gelelectrophoresis. Library preparation involved a TruSeq DNA PCR-free high-throughput library prep kit (Illumina) and Illumina genome sequencing was performed with a HiSeq1500 sequencer system 2 × 250 nt PE v2 HT (Illumina). The software snippy (https://github.com/tseemann/snippy) [67] was used for fast bacterial variant calling from NGS raw read data. The mapped data are available via BioProject: PRJNA669552. Detected SNPs in all triplicates were used for further analysis.

### 2.4. Quantification of Amino Acids and Organic Acids by HPLC or GC–MS

Extracellular amino acids and aromatic compounds were quantified by high-performance liquid chromatography (HPLC) (1200 series, Agilent Technologies Deutschland GmbH, Böblingen, Germany). The culture supernatants collected at different time points were centrifuged (20,200× *g*) for HPLC analysis. Separation was performed with a precolumn (LiChrospher 100 RP18 EC-5µ (40 × 4 mm), CS Chromatographie Service GmbH, Langerwehe, Germany) and a main column (LiChrospher 100 RP18 EC-5µ (125 × 4 mm), CS Chromatographie Service GmbH, Langerwehe, Germany). A mobile phase of buffer A (0.1% trifluoroacetic acid dissolved in water) and buffer B (acetonitrile) was used with a flow rate of 1 mL min^−1^. The following gradient was applied: 0–1 min 10% B; 1–10 min a linear gradient of B from 10% to 70%; 10–12 min 70% B; 12–14 min a linear gradient of B from 70% to 10%; 14–18 min 10% B [41]. The injection volume was 5 µL, and detection was performed with diode array detector at 210, 280, and 330 nm.

For GC–MS analysis, supernatants of strains C1* (pVWEx1), C1* (pVWEx1-*phe*), and C1*(pEKEx3-*cg2796-cg2797*) were analyzed after growth without or in the presence of 2.5 mM indole. Supernatants were collected after 16 h of cultivation and extracted with ethyl acetate. The ethyl acetate extract was washed three times with water to remove traces of CGXII medium components, followed by water removal using a Pasteur pipette plugged with silanized glass wool and Na_2_SO_4_. One microliter of each sample was injected into GC–MS. Samples were analyzed using a gas chromatograph (7890A, Agilent Technologies Deutschland) equipped with a 30 m × 0.25 mm × 0.25 µm film thickness column (DB-5, Phenomenex). Helium was used as the carrier gas and the flow rate was adjusted to 1 mL min^−1^ for GC–MS analysis. The injector was used in splitless mode and inlet temperature was set to 250 °C. The initial oven temperature was 45 °C for 1 min, and increased to 300 °C after 1 min at a rate of 10 °C min^−1^, which was held for 5 min at 300 °C. The GC was coupled to a Triple-Axis detector (5975C, Agilent Technologies Deutschland GmbH).

### 2.5. Protein Purification and Electrophoretic Mobility Shift Assay

After transformation of the pET16b-*cg3388* in *E. coli* BL21(DE3) transformants were grown at 37 °C in 500 mL LB medium with 50 µg mL^−1^ ampicillin to an OD_600_ of 0.5 before adding IPTG (0.5 mM) for induction of gene expression. After induction, cells were cultivated at 21 °C for an additional 4 h and were harvested by centrifugation at 4 °C. Pellets were stored at −20 °C. Crude extract preparation and protein purification via Ni-NTA chromatography was performed as described elsewhere [68]. The purified regulator protein Cg3388 was used for EMSA experiments without removing the N-terminal His-tag.

To analyze the physical protein–DNA interaction between the Cg3388 protein and their putative native target DNA, band shift assays were performed. The His-tagged Cg3388 protein was mixed in varying molar excess with 45 ng of PCR amplified and purified intergenic fragment between start codon of *cg3388* and *cg3387* (429 bp, using oligonucleotides cg3388_EMSA_F and cg3388_EMSA_R) in band shift buffer (50 mM Tris–HCl, 4% (*v*/*v*) glycerol, 50 mM KCl, 10 mM MgCl_2_, 0.5 mM EDTA, pH 7.5) in a total volume of 20 µL. The intergenic region was PCR-amplified and purified with NucleoSpin kit (MACHEREY-NAGEL GmbH & Co. KG, Düren, Germany). A 78 bp-fragment of the upstream region of *cg2228* was added in every sample as a negative control using oligonucleotides cg2228_EMSA_F and cg2228_EMSA_R. BSA (bovine serum albumin) was used as negative control. After 30 min of incubation at room temperature, gel shift samples were separated on a native 7.5% (*w*/*v*) polyacrylamide. Additionally, the binding affinity in the presence of myo-inositol and different aromatic compounds 1,2,4-trihydroxybenzene, hydroquinone, 1,2-dihydroxybenzene, 1,3-dihydroxybenzene, 2,4-dihydroxybenzoic acid, 34-dihydroxybenzoic acid (protocatechuate), indole, 6-hydroxyindole, 5-hydroxyindole, l-tryptophan, and 5-hydroxy-l-tryptophan as effector was analyzed by incubation of the protein with the effector under buffered conditions for 15 min at room temperature prior to the addition of the intergenic DNA region. Subsequently, the gel shift samples were separated on 7.5% acrylamide retardation gel at 100 V buffered in 44.5 mM Tris, 44.5 mM boric acid and 1 mM EDTA at pH 8.3. Staining of the DNA was achieved with ethidium bromide.

### 2.6. Iron Reduction Assay

The complex formation with the Fe^2+^-specific chelator bathophenanthroline disulfonic acid (BPS) was measured at 534 nm according to Müller et al. 2020 [69]. The kinetics of Fe^3+^ reduction was determined by an increase of absorbance. The iron reduction assay was performed in 25 mM Tris-HCL buffer (pH 7.4), and 0.42 mM FeCl_3_ (final concentration) was added from a stock in 10 mM HCl neutralized with 50 mM NaOH immediately before use; 19.5 μM PCA or indole was used. The reaction was started by the addition of 6.5 mM BPS and measured every 30 min. As control, reactions were measured without addition of PCA or indole.

### 2.7. Pseudo-Dipeptid Synthesis

For the synthesis of Indole-Ala-pseudo-Dipeptide, sodium hydride (60% suspension on paraffin oil, 0.1189 g, 2.96 mmol, 1.2 eq) was suspended in ice cold N,N-dimethylformamid (DMF) (4 mL) and a solution of Indole (1, 0.2792 g, 2.38 mmol, 1 eq) in DMF (8 mL) was added dropwise over 2 min and further stirred for 5 min at 0 °C. Boc-Ala-OH (2, 0.8505 g, 4.64 mmol, 1.9 eq) and 2-(1H-Benzotriazole-1-yl)-1,1,3,3-tetramethylaminium tetrafluoroborate (TBTU) (1.5922 g, 4.96 mmol, 2.1 equivalents) were dissolved in dimethylformamide (10 mL), followed by the dropwise addition of 4-methylmorpholin (NMM) (490 µL, 4.38 mmol, 1.8 equivalents) and stirred for 40 min at ambient temperature. The Boc-Ala-OH solution was then added to the ice-cold sodium hydride suspension over a period of 2 min. The ice bath was removed after 2.25 h and the reaction was further stirred for 1.3 h under ambient temperature. Afterwards the mixture was diluted with dichlormethane (DCM) (50 mL) and water (100 mL). The aqueous layer was extracted with DCM (3 × 50 mL), the organic layers were combined and washed with water (100 mL), saturated NaHCO_3_-solution (50 mL), aqueous HCl (1 M, 50 mL), water (100 mL) and saturated NaCl-solution (100 mL). The organic phase was dried over MgSO_4_ and the solvent was removed under reduced pressure. The crude product was used for the further reaction without purification. Boc-cleavage was performed under acidic conditions and cooling. Thereafter the crude of protected pseudo-Dipeptide was dissolved in DCM (5 mL), cooled to 0 °C with an ice bath, and treated slowly with HCl (4 M in dioxane, 4.6 mL, 1.9 eq). After 15 min the ice bath was removed, and the solution was stirred for 2.5 h at ambient temperature. Then the solvent was removed under reduced pressure, the residue was dissolved in water (50 mL) and DCM (50 mL). The aqueous layer was washed with DCM (2 × 50 mL, OP1), neutralized with sat. NaHCO_3_-solution (50 mL) and extracted with DCM (3 × 50 mL, OP2). OP2 was dried over MgSO_4_ and the solvent was removed under reduced pressure. The crude product was purified via reversed phase HPLC (water/ACN/0.1% trifluoracetic acid (TFA)) to receive the desired Indole-Ala-pseudo-Dipeptide (4, 0.1468 g, 0.48 mmol, 20%) as a colorless solid TFA-salt. The reaction mechanism and the ^1^H-NMR spectrum of the final product are shown in Supplementary Data Figure S1.

## 3. Results

### 3.1. Growth and Global Gene Expression Response of C. glutamicum to Extracellularly Added Indole

First, it was tested whether indole may serve as sole carbon or sole nitrogen source for growth of *C. glutamicum* strains WT and C1*. Indole (2 mM) was used to replace either the carbon equivalent of glucose (5.3 mM) or the nitrogen equivalent of the combined nitrogen sources urea and ammonium sulfate (0.7 and 1.31 mM, respectively). No growth was observed with indole as sole nitrogen or sole carbon source for 24 h, but both strains grew when afterwards either 220 mM glucose or 151 mM (NH_4_)_2_SO_4_ and 83 mM urea were added (data not shown). Thus, indole does neither support growth of *C. glutamicum* as sole carbon or nitrogen source.

**Figure 1:**
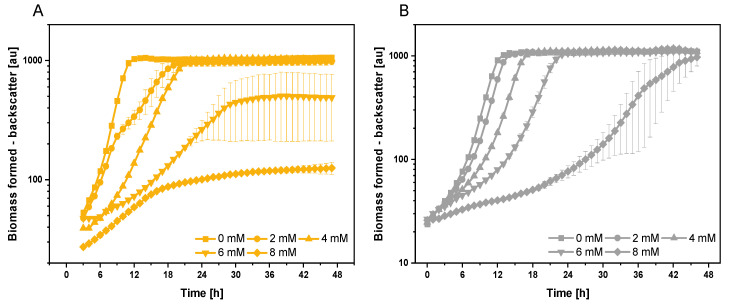
Growth of *C. glutamicum* WT (**A**) and C1* (**B**) in the presence of extracellularly added indole. Cultivation with the indicated indole concentrations was performed in the biolector cultivation system. Means and standard deviations of triplicate cultivations are shown.

To determine the growth response of *C. glutamicum* strains WT and C1* to indole as additive to glucose minimal medium, different indole concentrations (0 mM to 8 mM) were added to the medium before inoculation and growth in a biolector cultivation was monitored (Figure 1A). At 6 mM indole, for example, *C. glutamicum* WT showed a prolonged lag-phase, a growth rate decreased from 0.44 ± 0.00 to 0.14 ± 0.03 h^−1^ and it did not reach the same biomass concentration as the control without indole addition (Figure 1A). Growth of the genome-reduced *C. glutamicum* chassis strain C1* was also investigated since it grows as well as WT on glucose, but the response to indole was unknown (Figure 1B). Surprisingly, this strain showed a better growth performance in the presence of indole. For example, in the presence of 4 mM indole, C1* grew faster than WT (0.39 ± 0.00 h^−1^ as compared to 0.23 ± 0.00 h^−1^ for WT) and reached the same biomass concentration as the control without added indole (Figure 1B). The presence of 8 mM indole affected growth of both strains severely (Figure 1). While in a colony-forming assay no colony-forming units were observed after exposure to 8 mM indole for 1 h (Supplementary Data Figure S2), delayed growth in minimal media with 8 mM indole was observed to some extent for *C. glutamicum* WT and, although slow, strain C1* even grew to a comparable biomass concentration as without indole (Figure 1B).

To determine the gene expression changes of *C. glutamicum* strains WT to extracellularly added indole, a differential gene expression analysis was performed (Table 2). RNA was isolated from cells growing exponentially in the absence or in the presence of 2.5 mM indole. After confirmation of RNA integrity (RNA integrity number > 9) and the absence of DNA contamination, the prepared RNA samples from biological triplicates were pooled and sequenced. A total of 3.36 million reads generated from isolated and sequenced mRNA were obtained. The trimmed reads (70 pb after processing) were mapped to the genome of *C. glutamicum* WT. In total, 35 genes showed significantly increased RNA levels (adjusted *p*-value ≤ 0.01) in the presence of indole, while no gene showed decreased expression (Supplementary Data Figure S3A). Next, we chose a representative subset of genes for qRT-PCR analysis. As shown in Supplementary Data Figure S4A, qRT-PCR analysis of nine representative genes (*creF*, *cydB*, *phe*, *cg0405*, *irp1*, *porB*, *cg0591*, *thiC*, and *qcrR*) were performed. The pattern of differential gene expression determined in the RNAseq analysis was confirmed for all nine genes analyzed.

Among the genes that increased expression upon addition of 2.5 mM indole were phenol 2-monooxygenase gene *phe (cg2966)*, members of the DtxR regulon (e.g., *cg2796–2797*, *dps*, *htaB*, *htaC*, *ripA*), copper-related genes (e.g., *copB*, *copO*, *cg0464*, *cg3402*), and the operons for *p*-cresol catabolism and cytochrome bd (*cydABCD*) (Table 2). Since the *cydABCD* operon and the operon for *p*-cresol catabolism are absent from *C. glutamicum* C1*, which cannot utilize 2 mM *p*-cresol as sole carbon source (Supplementary Data Table S3), these expression changes were not considered further. However, while the differentially expressed *cydABCD* operon and the operon for *p*-cresol catabolism do not explain the effect of indole on growth of strain C1*, they might be relevant for the observed strain differences between WT and C1* regarding indole. This finding may guide future experiments to better understand the strain differences with respect to the response to indole. In this study, we used the wild type and a genome-reduced strain derived from the wild type by a series of confirmed deletions and focused on the shared traits with respect to the indole response.

Since the largest expression changes were observed for *phe* and the two DtxR regulated genes *cg2796* and *cg2797* coding for unknown proteins, it was tested if their deletion or overexpression affected growth of *C. glutamicum*. However, growth in the presence of indole was comparable to that of the parent strain (Supplementary Data Figure S5). Analysis of culture supernatants of *phe* overexpression, deletion, and complementation strains, grown in presence and absence of 2 mM indole, showed that indole concentrations decreased over time and could no longer be detected after 24 h. Instead, the extracellular tryptophan concentration increased up to 1 mM independent of the used strains, but only when indole was added. The *trpAB* encoded subunits of tryptophan synthase are believed to convert indole-3-glycerophospate to tryptophan without the release of indole. However, it is tempting to speculate that tryptophan synthase β subunit may convert indole and serine to tryptophan. Neither HPLC analysis nor GC–MS analysis of the strains overexpressing *phe* or *cg2796-cg2797* detected possible conversion products of indole (Supplementary Data Figure S6). Thus, neither overexpression nor deletion of *phe*, *cg2796*, and *cg2797* affected indole tolerance of *C. glutamicum.*

**Figure 2:**
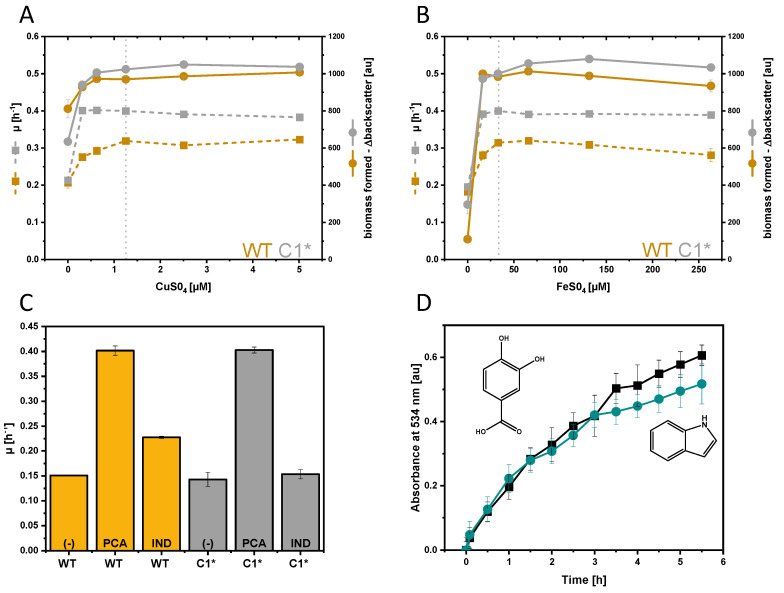
Growth of *C. glutamicum* WT (yellow) and C1* (gray) in the presence of varying CuSO_4_ (**A**) and FeSO_4_ (**B**) concentrations, with protocatechuate (PCA) or indole as iron chelators (**C**) and determination of iron reduction by indole and PCA as assayed with BPS (**D**). Maximal growth rates (dotted lines) and biomass concentrations (Δbackscatter, filled lines) are depicted as means and standard deviations of duplicate cultivations (**A**,**B**). Maximal growth rates of cultivations of WT and C1* without iron chelator (-) or with 195 µM PCA or indole as iron chelator are given as means and standard deviations of triplicates cultivations. The kinetics of Fe^3+^ reduction (**D**) were monitored using BPS as described in Material and Methods. Means and standard deviations of triplicates are shown.

Differentially expressed copper- and iron-related genes prompted us to investigate the effect of different iron and copper ion concentrations in the growth medium on the growth response of *C. glutamicum* WT and C1* to indole (Figure 2A,B). Copper and iron concentrations exceeding the normal media concentrations (1.25 and 36 µM, respectively, s. dotted lines in Figure 2A,B) did not increase the maximal growth rate or the maximal biomass formation in the presence of 4 mM indole. In addition, lowering of the medium copper and iron concentrations was not beneficial (Figure 2A,B).

Next, indole was tested as replacement of the common iron chelator protocatechuate (PCA) (Figure 2C). Precultures grown in CGXII minimal medium with 40 g L^−1^ glucose without addition of an iron chelator like PCA were used to inoculate main cultures containing either 195 µM PCA or 195 µM indole as iron chelators. Albeit WT reached a maximal growth rate of 0.23 ± 0.00 h^−1^ with indole, indole could not (fully) make up for PCA as an iron chelator that supported maximal growth rates of 0.40 ± 0.01 h^−1^ for WT and C1*. Based on the recent finding that PCA reduces Fe^3+^ to Fe^2+^ [69], a BPS-dependent iron reduction assay was performed. Surprisingly, 19.5 µM indole reduced Fe^3+^ to Fe^2+^ in a similar manner as 19.5 µM PCA (Figure 2D), while iron reduction was not observed without addition of indole or PCA. Thus, induction of iron-related genes by the extracellular addition of 2.5 mM indole (Table 2) may be due to its ability to reduce Fe^3+^ to Fe^2+^.

### 3.2. Transtriptome Analysis of C. glutamicum C1* in Response to Indole-Alanine Dipeptide

Since effects due to extracellularly added indole, e.g., due to reduction of medium Fe^3+^ to Fe^2+^ by indole (Figure 2D), may differ from increasing the intracellular indole concentration, a differential gene expression analysis was performed with *C. glutamicum* C1* grown in the absence or presence of 3 mM of the dipeptide indole-alanine. Indole-alanine dipeptide was prepared from indole and *tert*-butyloxycarbonyl protected l-alanine (Supplementary Data Figure S1). It is known that *C. glutamicum* takes up dipeptides fast and hydrolyzes them efficiently to the amino acid monomers [70]. While alanine is readily catabolized, indole is expected to accumulate in the *C. glutamicum* cell in a similar way as shown, e.g., for arginine upon addition of arginine-alanine dipeptide [71]. However, we did not measure the intracellular indole concentration, thus, it is only expected that the intracellular indole concentration was actually increased. Total RNA was isolated from cells growing exponentially in the absence or presence of 3 mM indole-alanine. In a parallel culture, no indole-alanine dipeptide could be detected by HPLC after 24 h cultivation, but 0.75 mM tryptophan (Supplementary Data Figure S1). After confirmation of RNA integrity and absence of DNA contamination, the prepared RNA samples were sequenced in three biological replicates. The total number of reads generated from isolated and sequenced mRNA was 10.5 million. The reads were trimmed to 70 bp before mapping to the genome of *C. glutamicum* C1*. In total, 37 genes showed increased expression (log2 fold change of RNA level ≥1.5; adjusted *p*-value < 0.01) in the presence of indole-alanine, whereas 6 genes showed decreased expression (log2 fold change of RNA level ≤ −1.5; adjusted *p*-value < 0.01; Table 3). The results obtained in the RNAseq analysis were validated by qRT-PCR for a subset of genes. The relative gene expression levels for four upregulated and four downregulated genes determined by qRT-PCR confirmed the pattern of their differential gene expression determined by RNAseq analysis (Supplementary Data Figure S4B).

Upon addition of indole-alanine, *phe*, *cg3195*, the gene coding for another monooxygenase, genes coding for citrate cycle and related enzymes (*sucCD*, *aceA*, *aceB*, *prpDBC1*, *prpDBC2*), regulator genes (*cg3303*, *znr* and *cg3127*), and *dps* encoding a starvation-induced DNA protecting protein showed increased RNA levels (Table 3). Reduced expression in the presence of indole-alanine was observed for 3-deoxy-7-phosphoheptulonate synthase gene *aroG*, RNase P gene *rnpA*, undecaprenol kinase gene *bacC*, *cg2719* coding for a putative enterochelin esterase, and two genes for putative membrane proteins (*cg2096*, *cg0286*). A common hallmark in the response to extracellular indole and to an increased intracellular indole concentration due to feeding indole-alanine peptide was increased expression of a subset of six genes: *cg0018* (coding for an uncharacterized membrane protein), *cg0464* (coding for a putative copper ion transporting P-type ATPase), *cg2962* (encoding a putative enzyme synthesizing extracellular polysaccharides), *cg3402* (coding for a copper chaperone), phenol oxygenase gene *phe*, and DNA protection gene *dps* (compare Table 2 and Table 3). This led us to the speculation that the response to indole may involve copper-dependent oxidation and transport processes.

### 3.3. Adaptive Laboratory Evolution for Increasing Indole Tolerance

**Figure 3:**
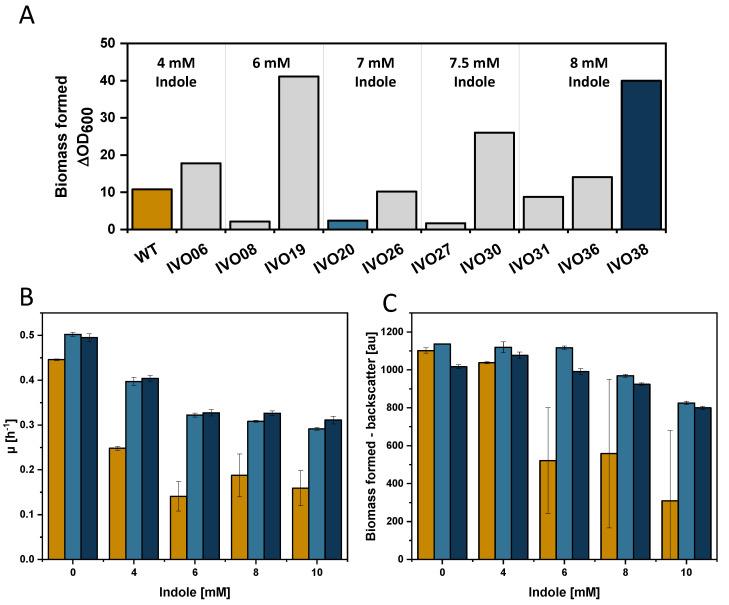
Adaptive laboratory evolution for fast growth in the presence of indole. The biomass formed after 24 h during the adaptive laboratory evolution at the indicated indole concentration is shown for selected transfers (**A**). Maximal growth rates (**B**) and biomass formation (**C**) of *C. glutamicum* WT (yellow), IVO20 (bright blue), and IVO38 (dark blue) with various indole concentrations shown as means and standard deviations of triplicate cultivations.

When higher indole concentrations were added to the culture medium, growth of the replicates differed (see large error bars, e.g., for WT and 6 mM indole in Figure 1A), which may indicate that growth accelerating mutations had occurred in one, but not all of the three replicates. This prompted us to isolate mutants with the ability of fast growth in the presence of indole and, therefore, an adaptive laboratory evolution (ALE) experiment was performed (Figure 3A). *C. glutamicum* WT cells were grown in the presence of indole for 38 serial transfers. After each serial transfer culture, a glycerol stock of the population was frozen and, in addition, a single colony was isolated on agar plates and frozen (named IVO20 to IVO38). The indole concentration added to fresh medium was stepwise increased (4 to 8 mM) in the serial transfers (Figure 3A). ALE strain IVO38 grew to an OD of 40 and was chosen for further analysis in addition to the intermediate ALE strain IVO20. In the presence of 4 to 10 mM, both ALE strains grew with higher growth rates to higher biomass concentrations than *C. glutamicum* WT (Figure 3B,C). Long lag phases were observed in the presence of 8 and 10 mM indole (data not shown). The biomass formed by IVO20 in the presence of 7 mM indole (Figure 3A) was low compared to that of IVO38 with 8 mM indole (Figure 3A), while both strains formed comparable biomass with 6 and 8 mM indole (Figure 3C). These data cannot be directly compared since the data in Figure 3A were taken from the ALE experiment, i.e., by inoculation by serial dilution, whereas the data shown in Figure 3C are from parallel growth experiments inoculated from defined precultures.

**Figure 4:**
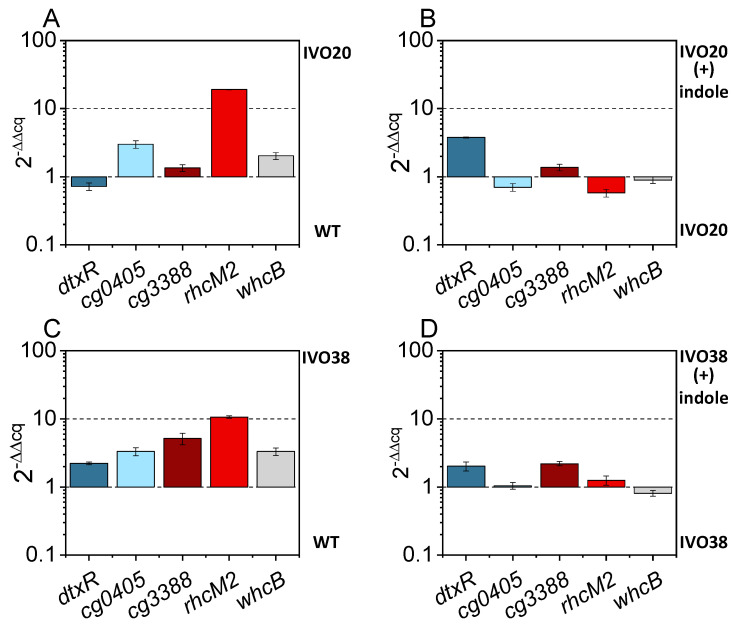
Results of qRT-PCR analysis for expression of *dtxR*, *cg0405*, *cg3388*, and *rhcM2* in the ALE strains IVO20 and IVO38 in the presence or absence of 4 mM indole. Comparisons of IVO20 and WT in the absence of indole (**A**), IVO20 (+) indole vs. without indole (**B**), IVO38 and WT in the absence of indole (**C**), and IVO38 (+) indole vs. without indole (**D**). The log2 fold change of the ΔΔCq value, using the reference gene *parA* is shown. Means and standard deviations of triplicate cultivations and independent performed qRT-PCRs are shown.

In order to identify possible mutations that caused improved growth of the ALE strains IVO20 and IVO38, their genomes were sequenced (Figure 4). As compared to the genome sequence of the parental *C. glutamicum* WT strain, ALE strain IVO20 possessed three nonsilent single-nucleotide polymorphisms (SNPs) and one insertion of 7 nucleotides (Table 4). The mutations led to a frameshift with premature stop codon (duplicate sequence of ACCGCAT at base 17 to 23) in 4-aminobutyrate aminotransferase gene *gabT* [72], to the amino acid exchanges R63L in WhcB, a stationary phase repressor of the gene for thioredoxin reductase, which restores oxidized proteins [73] and T8A in the DNA-binding helix-turn-helix motif of the regulator of iron metabolism DtxR [74]. Furthermore, the mutation of the translational start codon (ATG to ACG resulting in amino acid exchange M1T) of *cg3388*, which codes for a IclR-family transcriptional regulator, makes protein synthesis from this gene unlikely.

The genome of the ALE strain IVO38 showed an ~82 kbp deletion (~Δ*CGP3*; position 1776661-1986915), which was confirmed by PCR (Supplementary Data Figure S7). Excision of the prophage CGP3 is known to contribute to population heterogeneity in *C. glutamicum* [75]. Besides two synonymous mutations in cg1685 and the pyruvate kinase gene *pyk* [76] and one SNP in noncoding sequences, the genome of IVO38 contained five nonsilent SNPs in coding sequences (Table 4). Two SNPs altered the protein sequences of the aspartyl/glutamyl-tRNA^(Asn/Gln)^ amidotransferase subunit B GatB (D452G) and putative membrane protein Cg3132 (G69D). Amino acid exchange T2I in the regulator of oxidative stress response RosR may not affect the function of the protein. However, the corresponding base change on the DNA level alters one of the two RosR binding sites (binding site *rosRb* 5′-TAGGTGATATGA(C→T)AACAC-3′) affecting RosR autoregulation [54]. Two SNPs-affected genes mutated in IVO20 but led to different amino acid exchanges: R103H for DtxR and V310A for Cg3388. This argues that the single colony isolated from the grown population of the 20th transfer (IVO20) did not give rise to IVO38. (Note: transfers were done with aliquots of the grown culture, not with the single colonies isolated on agar plates from the grown cultures). Moreover, the finding of different mutations in their genes underscores the importance that DtxR and Cg3388 might have with respect to tolerance to indole.

In order to test if expression of *dtxR* and *cg3388* and their (putative) regulatory targets *cg0405* and *rhcM2* (*cg3386*), respectively, differs between *C. glutamicum* WT and the ALE strains IVO20 and IVO38, a qRT-PCR analysis was performed with RNA isolated from cells growing exponentially in the presence or absence of 4 mM indole (Figure 4). The RNA level of *dtxR* was higher in IVO38 than in WT (Figure 4C) and was increased in the presence of indole in IVO20 and IVO38 (Figure 4B,D). Expression of *cg0405*, which is repressed by DtxR, was comparable in the absence and presence of indole, but was higher in the ALE strains IVO20 and IVO38 than in *C. glutamicum* WT (Figure 4A,C). This may indicate derepression of *cg0405* as consequence of the amino acid exchanges T8A and R103H found in DtxR in the ALE strains IVO20 and IVO38, respectively. Cg3388 has not been characterized to date, but was shown here (s. below) to regulate the divergently transcribed operon *iolT2- rhcM2D2.*

The cg3388 RNA level was comparable in WT and IVO20, in which likely no Cg3388 protein is synthesized due to the SNP mutation of the translational start codon. However, in IVO38 (with SNP leading to Cg3388^G69D^) expression of cg3388 was higher than in WT (Figure 4C), and was further increased in the presence of indole (Figure 4D). More *rhcM2* RNA was found in IVO20 and IVO30 than in WT (Figure 4A,C), but indole did not further increase the *rhcM2* RNA level in the ALE strains (Figure 4B,D). This may be explained by derepression of *rhcM2* (and, possibly, the whole *iolt2- rhcM2D2* operon) due to absent or nonfunctional Cg3388. Reverse engineering revealed a major role of Cg3388^M1T^ in indole tolerance

**Figure 5:**
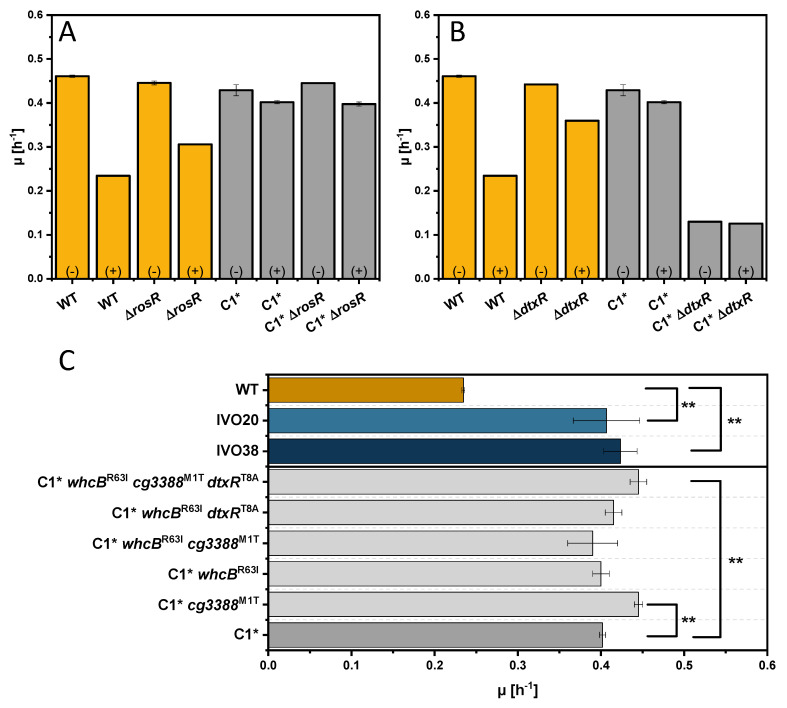
Growth comparison of *dtxR* (**A**) and *rosR* deletion strains (**B**) and reverse engineered strains (**C**) in presence of indole. Maximal growth rates of *rosR* deletions strains (**A**) and *dtxR* deletion strains (**B**) in absence (−) or presence of 4 mM indole (+) and of reverse engineered strains in the presence of 4 mM indole are shown as means and standard deviations of minimum of triplicates. Significance was determined based on a two-sided unpaired Student’s t-test (**: *p* < 0.05).

In order to test which mutations are causal for increased tolerance to indole, a number of mutants were constructed and characterized. As SNPs were detected for DtxR and RosR (Table 4) and members of their regulons were differentially expressed upon addition of indole and/or the dipeptide indole-alanine (Table 2 and Table 3), growth of the deletion strains WT, Δ*dtxR*, Δ*rosR*, C1*, C1*Δ*dtxR*, and C1*Δ*rosR* was analyzed in the absence or presence of indole. In the presence of indole, faster growth was observed for strain *dtxR* (0.36 ± 0.00) and *rosR* (0.31 ± 0.00) as compared to the wild type (0.23 ± 0.02), while growth in the absence of indole was comparable (Figure 5A,B). By contrast, deletion of neither *dtxR* nor *rosR* in C1* improved growth in the presence of indole (Figure 5A,B). The deletion of *dtxR* in C1* even reduced the growth rate independent of the presence or absence of indole. Thus, neither *dtxR* nor *rosR* mutations can explain the improved tolerance to indole observed in the ALE strains IVO20 and IVO38.

Next, the three SNPs detected in IVO20 were transferred to C1*, which lacks all prophages including CGP3 that also was absent from IVO38. Growth of *C. glutamicum* WT, the ALE strains IVO20 and IVO38, as well as of C1* and the derived strains C1*cg3388^M1T^, C1*whcB^R63L^, C1*cg3388^M1T^*whcB*^R63L^, C1*dtxR^T8A^*whcB*^R63L^, and C1*cg3388^M1T^dtxR^T8A^*whcB*^R63L^ was compared in the presence of 4 mM indole (Figure 5C). Among the C1* derived strains growth with indole was significantly faster for strains C1*cg3388^M1T^ and C1*cg3388^M1T^dtxR^T8A^*whcB*^R63L^ (Figure 5C). Thus, Cg3388^M1T^ plays a major role in indole tolerance.

### 3.4. Investigation of the Transcriptional Regulator Cg3388

**Figure 6:**
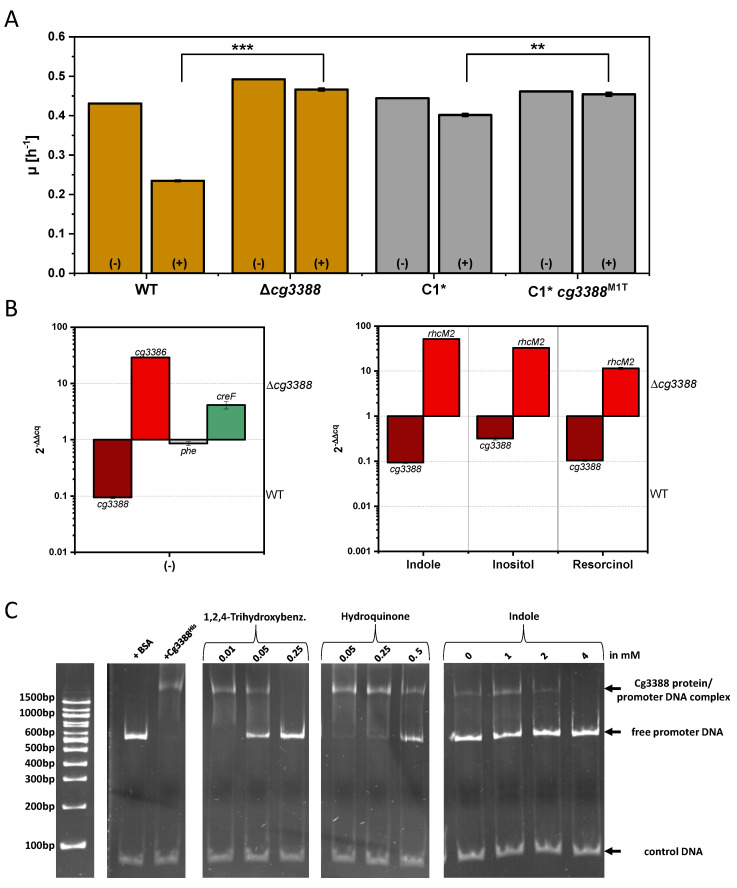
Indole-dependent effect on Cg3388. (**A**) Growth rates of strains WT, Δ*cg3388*, C1*, and C1* *cg3388*^M1T^ in CGXII minimal medium with 40 g L^−1^ glucose as carbon source in absence (−) and presence of 4 mM indole (+) are given as means and experimental imprecision of duplicates. Significance was determined based on a two-sided unpaired Student’s t-test (**: *p* < 0.05); *** *p* < 0.01). (**B**) qRT-PCR analysis of WT and WT Δ*cg3388* cells grown in presence of 2.5 mM indole, inositol, or resorcinol is shown for *cg3388* (dark red), *rhcM2* (light red), *phe* (gray), and *creF* (green) as means and standard deviations of triplicate cultivations. (**C**) Electrophoretic mobility shift assays (EMSA) with His-tagged Cg3388 (153 fold molar excess) and the intergenic region (45 ng) between cg3388 and *iolT2* with addition of 1,2,4-trihydroxybenzene (0.01–0.025 mM), hydroquinone (0.05–0.05 mM), or indole (1–4 mM) are shown.

Two SNPs in cg3388 leading to amino acid changes were found: M1T and G69D. The SNP in the translational start codon (M1T) may result in the complete absence of Cg3388 protein, while the effect of the amino acid change G69D is not known. Therefore, we deliberately chose to delete cg3388 as we consider this the most clear cut genetic modification. Strain Δ*cg3388* was constructed to test if deletion of *cg3388* improves indole tolerance in a similar manner as *cg3388*^M1T^. Indeed, in the presence of indole the *cg3388* deletion mutant grew faster than WT (0.47 ± 0.00 vs. 0.23 ± 0.00 h^−1^), which was comparable to the growth rate observed with C1*cg3388^M1T^ (0.45 ± 0.00 h^−1^; Figure 6A). This result corroborated the finding that Cg3388 plays a major role in the response of *C. glutamicum* to indole.

Due to similarities to other IclR-type transcriptional regulators that regulate adjacent, divergently transcribed operons, we tested if Cg3388 regulates the divergently transcribed operon *iolT2-rhcM2D2*. A qRT-PCR analysis of WT and Δ*cg3388* grown without or with 2.5 mM indole revealed increased RNA levels of *rhcM2*. This finding is commensurate with a role of Cg3388 as repressor of *rhcM2* and, by inference, likely the *iolT2-rhcM2D2* operon. UniProtKB analysis predicts an N-terminal helix-turn-helix DNA binding domain (HTH iclR-type) at position 20–81 and a C-terminal effector binding domain (IclR-ED) at position 82–266 for Cg3388 (Q8NL89), a domain structure typical for repressor proteins. As *rhcM2* and *rhcD2* codes for maleylacetate reductase and catechol 1,2-dioxygenase enzymes involved in resorcinol catabolism [23] and as IolT2 imports inositol into the *C. glutamicum* cell [77], the effect of addition of resorcinol and inositol was tested, but RNA levels of *cg3388* and *rhcM2* were comparable to indole addition (Figure 6B).

In addition, the effect of *cg3388* deletion on the gene expression of *phe* and *creF*, which showed increased RNA levels under conditions with external and/or internal added indole, was analyzed. While *phe* RNA levels were not affected by deletion of *cg3388*, *creF* showed increased RNA levels in the absence of Cg3388.

In order to test if Cg3388 protein physically interacts with target promoter DNA, an electrophoretic mobility shift assay (EMSA) was performed (Figure 6C). Purified His-tagged Cg3388 protein was incubated with the PCR amplified intergenic DNA region between the start codons of *cg3388* and *iolT2*. A band shift was observed with His-tagged Cg3388 protein, but not with BSA used as negative control protein. Thus, Cg3388 binds to the intergenic region between its own gene and the *iolT2- rhcM2D2* operon.

For identification of effectors of Cg3388, different compounds were added during the incubation of His-tagged Cg3388 protein with target DNA. The binding of Cg3388 to target DNA was hardly affected by the addition of 5 mM of inositol, resorcinol (1,3-dihydroxybenzene), 1,2-dihydroxybenzene, 2,4-hydroxybencoic acid, PCA (3,4-dihydroxybenzoic acid), 6-hydroxyindole, 5-hydroxyindole, l-tryptophan, and 5-hydroxy-l-tryptophan (data not shown). By contrast, 1,2,4-trihydroxybenzene, hydroquinone (1,4-dihydroxybenzene), and indole interfered with binding of Cg3388 to its target DNA at concentrations exceeding 0.05, 0.5, and 4 mM, respectively (Figure 6C). Thus, Cg3388 responds to 1,2,4-trihydroxybenzene, hydroquinone, and indole as effector molecules that may function as inducers of *cg3388* and the *iolT2-rhcM2D2* operon. Cg3388 was named IhtR for 1,2,4-trihydroxybenzene, hydroquinone, and indole responsive repressor. Since 1,2,4-trihydroxybenzene affected DNA binding of Cg3388 at the lowest concentration (0.05 mM) and as maleylacetate reductase RolM and catechol 1,2-dioxygenase RolD are homologs of RhcD2 and RhcM2 converting 1,2,4-trihydroxybenzene to β-ketoadipate, 1,2,4-trihydroxybenzene is most likely the main physiological effector of Cg3388/IhtR. However, since hydroquinone and indole affected Cg3388/IhtR in vitro at physiologically relevant concentrations they may also play a role in *C. glutamicum* in vivo.

## 4. Discussion

The response of *C. glutamicum* to indole, which was shown to reduce Fe^3+^ to Fe^2+^ in the culture medium, is governed by increased expression of iron and copper metabolism genes, of phenol 2-monooxygenase gene *phe*, and the *p*-cresol catabolic *cre* operon. Deletion of genes encoding the regulator of oxidative stress response RosR, the iron response regulator DtxR, and Cg3388/IhtR improved indole tolerance.

Extracellular indole affected expression of genes of iron homeostasis as it reduced Fe^3+^ to Fe^2+^ (Figure 2D) and deletion of the iron homeostasis repressor *dtxR* improved growth in the presence of indole (Figure 5A). ALE strain IVO38 lacked prophage CGP3 and possessed a point mutation in *dtxR* (Table 4). Excision of CGP3 is known to be prevalent in *dtxR* mutants, possibly triggered by DNA damages caused by elevated intracellular iron concentrations [75]. The fact that *dtxR* is not autoregulated nor known to be regulated by other transcriptional regulators [78] may explain that *dtxR* mutations occur relatively often. Thus, improved indole tolerance of *C. glutamicum* required upregulation of DtxR targets that include intracellular iron storage proteins, high-affinity iron uptake systems, redox-stress resistance systems, and iron-containing proteins [53,78]. Growth in the presence of indole, however, was not improved by increasing the iron concentration in the culture medium (Figure 2B). Uptake of copper ions depends on iron-containing proteins as it is proposed that copper ions are either transported with the help of Cg1883 and Cg0520 to the cytochrome complex and/or with the help of CopC and CtiP, CopC and Cg1883 [74,79,80]. This may explain that indole also triggered the copper starvation response (increased RNA levels of *copO*, *copB*, and *copC*) in addition to the DtxR regulon (e.g., cytochrome *bd* genes *cydABCD*). Notably, when the intracellular indole concentration was increased by feeding the dipeptide indole-alanine, only two genes (*dps* and *cg0405*) of the DtxR regulon were induced (Table 3). Apparently, the presence of indole in the growth medium is required to trigger the DtxR regulon, likely by chemical reduction Fe^3+^ to Fe^2+^ by indole.

The response to the addition of the aromatic compound indole elicited increased expression of genes belonging to three pathways: phenol 2-monooxygenase (*phe*), the *cre* operon for catabolism of *p*-cresol, and the transcriptional regulator Cg3388/IhtR. Strain C1* lacks the *cre* operon and cannot use cresol as sole carbon source (Supplementary Data Table S3). Notably, as strain C1* possesses intact copies of *dtxR*, *rosR*, and *cg3388*, the absence of the *cre* operon may contribute to the higher indole tolerance of C1* as compared to WT. In addition, or alternatively, the absence of CGP3 and the *cydABCD* cluster from C1* may explain this strain difference. Cresol degradation is initiated by phosphorylation of the hydroxyl group catalyzed by 4-methylbenzyl phosphate synthase [25,26]. However, indole does not carry a hydroxyl group and has a heterocyclic structure consisting of one benzene ring and one pyrrole ring. This led us to speculate that indole elicits increased expression of the *cre* operon as it may be similar to the unknown inducer of this operon. On the other hand, we observed increased *creF* RNA levels upon deletion of *cg3388* (Figure 6B). Thus, Cg3388/IhtR may be involved directly or indirectly in the regulation of the *cre* operon. If C1* grows faster in the presence of indole because induction of the *cre* operon results in a metabolic burden or if an enzyme encoded by the *cre* operon converts indole to a more toxic compound remains to be studied. The latter may be less likely since we did not find indole degradation products in the supernatants (Supplementary Data Figure S6).

*Phe* encodes phenol 2-monooxygenase, which oxidizes phenol to catechol in an NADPH-dependent manner [81,82]. While it is known that the transcriptional AraC-type regulator PheR activates *phe* expression in the presence of phenol, *phe* is not expressed in the presence of glucose or absence of PheR [75,76]. Notably, *phe* expression increased upon addition of indole extracellularly as well as due to increased intracellular levels upon feeding indole-alanine dipeptide (Table 3 and Supplementary Data Figure S4). However, overexpression or deletion of *phe* did not improve indole tolerance and HPLC analysis revealed decreasing indole and increasing tryptophan concentrations, whereas no further aromatic compounds were detected by HPLC or GC–MS analysis (Figure S6). Increased *phe* expression upon increasing indole concentrations may be due to regulation as we speculate that indole might serve as inducer of PheR.

Adaptive laboratory evolution to increase indole tolerance led to the discovery of Cg3388/IhtR as indole, hydroquinone, and 1,2,4-trihydroxybenzene responsive transcriptional regulator repressing transcription of the divergently transcribed *iolT2-rhcM2D2* operon. IolT2 and IolT1 are known to import inositol, glucose, and fructose into the *C. glutamicum* cell [77,83], while import of xylose has only been found for IolT1 [84]. Transcription of *iolT1* and the large *iol* operons are repressed by IolR, which is responsive to myo-inositol or a myo-inositol degradation product [85]. By contrast, inositol-dependent regulation of *iolT2* has not yet been observed and inositol was shown here not to interfere with DNA binding of Cg3388/IhtR (see above). The *iolT2-rhcM2D2* operon also encodes catechol 1,2-monooxygenase and maleylacetate reductase enzymes. Paralogs are encoded by the *rol* operon (cg1112-cg1115), which is negatively regulated by RolR and required for catabolism of resorcinol and 2,4-dihydroxybenzoate [24,30]. Although the *iolT2-rhcM2D2* operon is not required for growth with resorcinol, it is, however, transcribed in the presence of resorcinol [23]. Resorcinol did not function as inducer of Cg3388/IhtR (Figure 6), but the intermediate of resorcinol degradation 1,2,4-trihydroxybenzene was the most effective Cg3388/IhtR inducer (≥0.05 mM) (Figure 6). Possibly, Cg3388/IhtR is important to regulate the *iolT2-rhcM2D2* operon, which is derepressed in the presence of 1,2,4-trihydroxybenzene or aromatic compounds other than the inducers of RolR resorcinol and hydroxyquinol that lead to 1,2,4-trihydroxybenzene. Indole only induced the *iolT2-rhcM2D2* operon, but not the *rol* operon; thus, it is not an inducer of RolR. Induction of the *iolT2-rhcM2D2* operon by indole may be a metabolic burden, slowing growth in its presence or indole may be converted to a growth inhibitory compound by catechol 1,2-monooxygenase RhcM2 and/or maleylacetate reductase RhcD2. In the latter case, this compound may be synthesized intracellularly, but we did not obtain evidence for its accumulation in the culture medium. The nature of this putative indole-derived inhibitor remains elusive.

## Figures and Tables

**Table 1:** Bacterial strains used in this study.

Strains	Description	Source
*Corynebacterium glutamicum*		
WT	*C. glutamicum* wild type ATCC13032	ATCC
C1*	Genome-reduced chassis strain derived from WT	[52]
Δ*dtxR*	WT containing *dtxR* deletion	[53]
Δ*rosR*	WT containing ros*R* deletion	[54]
Δ*cg3388*	WT containing *cg3388/ihtR* deletion	[55]
C1* Δ*dtxR*	C1* containing *dtxR* deletion	This study
C1* Δ*rosR*	C1* containing ros*R* deletion	This study
IVO20	Strain evolved from WT after 20 transfers in the presence of indole	This study
IVO38	Strain evolved from WT after 38 transfers in the presence of indole	This study
C1* *cg3388*^M1T^	C1* with SNP in *cg3388/ihtR*, resulting in amino acid exchange M1T	This study
C1* *whcB*^R63L^	C1* with SNP in *whcB*, resulting in amino acid exchange R63L	This study
C1* *cg3388*^M1T^ dtxR^T8A^	C1* with SNP in *cg3388/ihtR* and *dtxR*, resulting in amino acid exchange M1T and T8A	This study
C1* *cg3388*^M1T^ *whcB*^R63L^	C1* with SNP in *cg3388/ihtR* and *whcB*, resulting in amino acid exchanges M1T and R63L, respectively	This study
C1* *cg3388*^M1T^ dtxR^T8A^ *whcB*^R63L^	C1* with SNPs in *cg3388/ihtR*, *dtxR and whcB*, resulting in amino acid exchanges M1T, T8A and R63L, respectively	This study
C1* (pVWEx1)	C1* carrying pVWEx1	This study
C1* (pVWEx1-*phe*)	C1* carrying pVWEx1-*phe*	This study
C1* Δ*phe* (pVWEx1)	C1* containing *phe* deletion, carrying pVWEx1	This study
C1* Δ*phe* (pVWEx1-*phe*)	C1* containing *phe* deletion, carrying pVWEx1-*phe*	This study
C1* (pEKEx3)	C1* carrying pEKEx3	This study
C1* (pEKEx3-*cg2796-cg2797*)	C1* carrying pEKEx3-*cg2796-cg2797*	This study
C1* Δ*cg2796-cg2797* (pEKEx3)	C1* containing *cg2796-cg2797* deletion, carrying pEKEx3	This study
C1* Δ*cg2796-cg2797* (pEKEx3-*cg2796-cg2797*)	C1* containing *cg2796-cg2797* deletion, carrying pEKEx3-*cg2796-cg2797*	This study
*Escherichia coli*		
S17-1	*recA pro hsdR RP4-2-Tc::Mu-Km::Tn7*	[56]
DH5α	*F-thi-1 endA1 hsdr17(r-*, *m-) supE44 1lacU169 (Φ80lacZ1M15) recA1 gyrA96*	[49]
BL21 (DE3)	*F– ompT gal dcm lon hsdSB(rB–mB–) λ(DE3[lacI lacUV5-T7p07 ind1 sam7 nin5])[malB+]K-12(λS)*	[57]

**Table 2:** List of genes differentially expressed in the presence of 2.5 mM indole. *C. glutamicum* WT was cultivated in CGXII minimal medium with 40 g L^−1^ glucose in the presence of 2.5 mM indole and cells were harvested during exponential growth (OD_600_ 4). Loci, genes, gene products, and Log2 fold changes of RNA levels are shown for genes with statistically significant expression changes (adjusted *p*-value ≤ 0.01) with a log2 fold change >1.5 or <−1.5.

Locus	Gene	Gene Product	Ind/-
cg0018		uncharacterized membrane protein	4.09
cg0405		ABC-type Fe^3+^-siderophore transport systems	2.57
cg0470	*htaB*	heme binding protein	3.12
cg0471	*htaC*	heme binding protein	2.91
cg0637	*creC*	benzylaldehyde dehydrogenases	2.85
cg0638	*creD*	phosphohydrolase	3.11
cg0639	*creE*	class I P450 system subunit	3.59
cg0640	*creF*	class I P450 system subunit	3.81
cg0641	*creG*	NAD^+^-dependent 4-hydroxybenzyl alcohol dehydrogenase	3.01
cg0642	*creH*	4-methylbenzyl phosphate synthase subunit	3.60
cg0644	*creI*	4-methylbenzyl phosphate synthase subunit	3.95
cg0645	*creJ*	class I P450 system subunit	3.96
cg1120	*ripA*	AraC-type DNA-binding domain-containing proteins	2.47
cg1152	*seuB*	acyl-CoA dehydrogenases	3.20
cg1298	*cydC*	ATP-binding/permease protein	3.69
cg1299	*cydD*	ATP-binding/permease protein	3.77
cg1300	*cydB*	cytochrome bd-type quinol oxidase, subunit 2	3.84
cg1301	*cydA*	cytochrome bd-type quinol oxidase, subunit 1	3.72
cg1773	*ctaB*	polyprenyltransferase	2.45
cg1881		predicted iron-dependent peroxidase	3.15
cg1883		uncharacterized secreted protein	3.15
cg1884	*copC*	membrane-bound copper chaperone	3.08
cg1930		hypothetical protein	3.46
cg1931		hypothetical protein	2.72
cg2678		ABC-type transporter. periplasmic component	2.44
cg2796		uncharacterized protein involved in propionate catabolism	6.87
cg2797		uncharacterized ACR	6.29
cg2962		putative enzyme synthesing extracellular polysaccharides	3.69
cg2966	*phe*	putative phenol 2-monooxygenase	6.14
cg3280		uncharacterized secreted protein	2.70
cg3281	*copB*	cation transport ATPases	2.67
cg3286		hypothetical protein	3.09
cg3287	*copO*	multicopper oxidase	3.11
cg3289		thioredoxin-like protein	3.82
cg3327	*dps*	starvation-inducible DNA-binding protein	4.40

**Table 3:** List of genes differentially expressed in the presence 3 mM indole-alanine dipeptide. The *C. glutamicum* strain C1* was cultivated in CGXII minimal medium with 40 g L^−1^ glucose in presence 3 mM indole-alanine; cells were harvested during exponential phase (OD_600_ 6). Loci, genes, gene products, and Log2 fold changes of RNA levels are shown for genes with statistically significant expression changes (adjusted *p*-value ≤ 0.01) in at least one comparison with a log2 fold change > 1.5 or < −1.5.

Locus	Gene	Gene Product	Ind-Ala/-
cg0012	*ssuR*	transcriptional activator of sulfonate(ester) utilization	1.84
cg0018		uncharacterized membrane protein	5.36
cg0120		putative hydrolase	1.66
cg0175		putative secreted protein	1.85
cg0192		hypothetical protein	1.97
cg0286		putative membrane protein	−2.23
cg0464		putative Cu^2+^ transporting P-type ATPase	1.59
cg0569		cation transporting ATPase	2.05
cg0759	*prpD2*	methylcitrate dehydratase, involved in propionate catabolism	2.38
cg0760	*prpB2*	methylisocitrate lyase, involved in propionate metabolism	2.53
cg0762	*prpC2*	methylcitrate synthase, involved in propionate catabolism	2.57
cg0796	*prpD1*	putative (2-methyl) citrate dehydratase	1.65
cg0797	*prpB1*	putative (methyl)isocitrate lyase	1.82
cg0798	*prpC1*	putative (methyl)citrate synthase	1.76
cg1279		putative secreted protein	2.05
cg1327		putative transcriptional regulator, Crp-family	1.53
cg1328		putative heavy-metal ion transporting P-type ATPase	1.72
cg1393		putative acetyltransferase, GNAT family	1.78
cg1470		hypothetical protein	2.58
cg1471		hypothetical protein	1.51
cg1635		putative membrane protein	1.51
cg1710	*bacA*	undecaprenol kinase	−1.77
cg2096		putative membrane protein	−1.59
cg2391	*aroG*	3-deoxy-7-phosphoheptulonate synthase	−1.54
cg2500		putative transcriptional regulator. ArsR-family	1.59
cg2559	*aceB*	malate synthase	1.79
cg2560	*aceA*	isocitrate lyase	2.34
cg2719		putative enterochelin esterase	−1.6
cg2836	*sucD*	succinate-CoA ligase (ADP-forming), alpha subunit	2.06
cg2837	*sucC*	succinate-CoA ligase (ADP-forming), beta subunit	2.23
cg2962		putative enzyme synthesing extracellular polysaccharides	2.12
cg2966	*phe*	putative phenol 2-monooxygenase	2.08
cg3169	*pck*	phosphoenolpyruvate carboxykinase (GTP)	2.00
cg3195		putative flavin-containing monooxygenase	1.60
cg3202	*farR*	transcriptional regulator. GntR-family	1.88
cg3226		putative MFS-type L-lactate permease	2.05
cg3303		putative PadR-family transcriptional regulator	2.26
cg3327	*dps*	starvation-inducible DNA-binding protein	1.85
cg3367		ABC-type multidrug transport system, ATPase	1.72
cg3402		copper chaperone	2.03
cg3431	*rnpA*	ribonuclease P	−1.70
cg4019		hypothetical protein	3.57
cg4028		hypothetical protein	2.05

**Table 4:** Single-nucleotide polymorphisms (SNPs) determined by whole-genome sequencing of ALE strains IVO20 and IVO38. Nonsilent SNPs and insertions found in the coding sequence of the strains IVO20 and IVO38 are given with the corresponding loci, gene names, gene products, and the resulting amino acid substitutions.

Locus	Gene Name	Amino Acid Exchange	Gene Product
cg0695	*whcB*	R63L in IVO20	Stationary phase repressor protein
cg2103	*dtxR*	T8A in IVO20	Transcriptional iron homeostasis repressor
cg3388	*-*	M1T in IVO20	Putative transcriptional regulator. IclR-family
cg0566	*gabT*	insertion in IVO20 (ACCGCAT pos. 17 to 23)	4-aminobutyrate aminotransferase
cg1324	*rosR*	T2I in IVO38	Transcriptional regulator of oxidative stress response
cg1420	*gatB*	D452G in IVO38	Glutamyl-tRNA (Gln) amidotransferase. subunit B
cg2103	*dtxR*	R103H in IVO38	Transcriptional iron homeostasis repressor
cg3132	*-*	V310A in IVO38	Putative membrane protein
cg3388	*-*	G69D in IVO38	Putative transcriptional regulator. IclR-family

## Data Availability

The mapped genome sequencing data is available via BioProject: PRJNA669552 and the transcriptomic data is available via NCBI GEO accession identifiers GSE159887 and GSE159888.

## Supplementary Materials

Supplementary materials can be found at https://www.mdpi.com/2076-2607/8/12/1945/s1.
