# Supplementary material for: Physiological Response of Corynebacterium glutamicum to Indole
Source: Microorganisms. 2020 Dec 8;8(12):1945. doi: 10.3390/microorganisms8121945 (PMC7764795; doi:10.3390/microorganisms8121945)
Supplement: Supplementary file 1 [file microorganisms-08-01945-s001.zip › Supplementary Data_indole response/Supplementary Data Table S1 to S3.pdf]

Table S1: Oligonucleotides used in this study. Ribosomal binding sites are in bold and plasmid binding regions of Gibson oligonucleotides are underlined.

Name	Oligonucleotide sequence (5′ to 3′)
**Used for plasmid construction. deletion. and integration**	
Δphe_US_F	CCTGCAGGTCGACTCTAGAGGAACGCCAACTAAGTCGAATCTTC
Δphe_US_R	CTGTGCCTTAGTTCGCAAACTGCATGTCTGGTCCTTTC
Δphe_DS_F	GCAATGACCGAAGCGCCTTAAATACCAGGTCAG
Δphe_DS_R	GAATTCGAGCTCGGTACCCGGGGAACTCACTGAAGAAACCGGCAG
Δphe_G_F	CCGAAAGTGACTTACAAGCGTC
Δphe_G_F	CGATAAAATAGCTGGCTAGACC
*phe*_P_F	CCTGCAGGTCGACTCTAGAG**GAAAGGAGGCCCTTCAG**ATGCAGTTTCATTATGAAGGATACGC
phe_P_R	TGAATTCGAGCTCGGTACCCGGGTTAGTTCGCGTTGATTAGCTGATG
Δ*cg2796-cg2797*_US_F	GCCTGCAGGTCGACTCTAGAGGTCACCTTCCCCCAAAAATGATG
Δ*cg2796-g27976*_US_R	GGTATTTAAGGCGCTTCGGTCATTGCAATCCTATC
Δ*cg2796-g27976*_DS_F	GCAATGACCGAAGCGCCTTAAATACCAGGTCAG
Δ*cg2796-g27976*_DS_R	GAATTCGAGCTCGGTACCCGGGGAACTCACTGAAGAAACCGGCAG
Δ*cg2796-g279766*_G_F	CAGAGCCATTGCGTCCTTG
Δ*cg2796-g27976*_G_R	CCGAAACGGCGACAACAT
*cg2796-g27976*_P_F	CATGCCTGCAGGTCGACTCTAGAG**GAAAGGAGGCCCTTCAG**ATGACCGAATCGCAAGATCTC
*cg2796-g27976*_P_R	GAATTCGAGCTCGGTACCCGGGTTAAGGCGCAGGGTTGATTACTC
SNP_cg3388_F	CCTGCAGGTCGACTCTAGAGGTCCGAGTTCTTGTGCCATG
SNP_cg3388_R	GAATTCGAGCTCGGTACCCGGGGTTTCCTTCAACGTGTCCCG
cg3388_G_F	CCAAAGGCTGCAGCGAAAAC
cg3388_G_R	GCTCACCTACTTTTGCGGCG
cg3388_P_F	GCGGCCATATCGAAGGTCGTCATATGCAAACCATTCAGCTACTCACC
cg3388_P_R	GTTAGCAGCCGGATCCTCGAGCATTTAAACCACAATGCTCAGAGGGGTTAC
cg3388_EMSA_F	CATGTTGTGTCCTTTTATCTTTGATGAAGTCAC
cg3388_ EMSA _R	CATATTTGATCCGTTTTTCTAAAGGTTGTTTTTGATTTTG
cg2288_ EMSA _F	
cg2288_ EMSA _R	
SNP_dtxR_F	CCTGCAGGTCGACTCTAGAGCCGTTTGCGCTACGGCC
SNP_dtxR_R	GAATTCGAGCTCGGTACCCGGGCGTTGTGGGTGATCACGACC
ΔdtxR_F	CCTGCAGGTCGACTCTAGAGGAGGAACTCGCAGAAGAGTCC
ΔdtxR_R	GAATTCGAGCTCGGTACCCGGGGTTACTTCACGGCACATCTCC
dtxR_G_F	CATCCGCCTCCCAGTCCAC
dtxR_G_R	CTCAGGGGTCCAACCCAG
SNP_whcB_F	CCTGCAGGTCGACTCTAGAGCTAAAAGTTTAATAATAAGACAAGTATAAAGCCCTCAT
SNP_ whcB_R	GAATTCGAGCTCGGTACCCGGGCTCTGAAGGCTTACGCCGAAG
ΔrosR_F	CCTGCAGGTCGACTCTAGAGCAGAAACCAGAACGCCACCG
ΔrosR_R	GAATTCGAGCTCGGTACCCGGGCTAACTCCAGTCGTTCCCATCAC
rosR_G_F	CTCGACGCCAAAAGCCGC
rosR_G_R	GCCAAGCAGACCACCGAG
**Used for qRT-PCR**	
RosR_F	GCATGATCTTGTCTGCAACC
RosR_R	CCACTAAGCCCTTTTTGTCC
Cg1322_F	AGAACGAAAACGAAGGCAC

Cg1322_R	TCAGAAACCAGAACGCCAC
Cg1150_F	TTTCAACTTCCCCGTCCTC
Cg1150_R	CAGCCTTGTTGTTCCACTC
DtxR_F	TAGCAGAACGCCTCCTTAC
DtxR_R	ATCCAAACCGATTTCGCC
Cg0405_F	ACGGACAAACCGCTTCAATG
Cg0405_R	CAACCCACAGTTCGGAAATC
Cg3388_F	GCAAACCATTCAGCTACTCAC
Cg3388_R	GAGCCAGCCTAATTTTTCCC
Cg3386_F	AACTCACTCGCATTCAACC
Cg3386_R	CGACATTTCTCGTTCACCC
WhcB_F	ACATTGCCTCACCAGCTTCC
WhcB_R	TCTGAAAGTCCGCCCCATAC
GatB_F	AATCGCATACGACGGCTAC
GatB_R	GACAACCTCAATCAAAGGGAC
Cg3132_F	GAACAATGCGACGATGAATAAG
Cg3132_R	AACCAACCAAAACCAACCC
CreF_F	CACCATCGAGGCGACTGTTG
CreF_R	CCATGCCTTCGGTGACCTTG
*Phe*_F	AGGATGAAGCCTGACTATGG
Phe_R	TGGTTAGCCTGCTTACCTTG
ParB_F	GAGCGTAGTACTCACACTGG
ParB_R	GCTGGCGCAGAAATTGAATT
**Used for CGP3 verification**	
CGP3_A_F	CTGAGCTAAACGCGCGGG
CGP3_B_R	CAAACGCGTGTTTTTTAACCC
CGP3_C_F	CCCGGTTTCATTAAGAATCATGTCCAC
CGP3_D_R	CATTCTTGGGCGCTTCCTG

Table S2 List of plasmids used in this study

Plasmids	Description	Source
pK19*mobsacB*	Km^R; *E. coli*/*C. glutamicum* shuttle vector for construction of insertion and deletion mutants in *C. glutamicum* (pK19 *oriV$_{Ec}$* *sacB* *lacZα*)	[58]
pK19*mobsacB* -Δ*rosR*	pK19*mobsacB* with a construct for deletion of *rosR* (cg1324)	This study
pK19*mobsacB* -Δ*dtxR*	pK19*mobsacB* with a construct for deletion of *dtxR* (cg2103)	This study
pK19*mobsacB* -Δphe	pK19*mobsacB* with a construct for deletion of *phe* (cg2966)	This study
pK19*mobsacB* -Δ*cg2796-cg2797*	pK19*mobsacB* with a construct for deletion of *cg2796-cg2797*	This study
pK19*mobsacB* -*whcB*^{R63L}	pK19*mobsacB* with a construct for the amino acid exchange R63L in *whcB* (cg0695)	This study
pK19*mobsacB* -*cg3388*^{M1T}	pK19*mobsacB* with a construct for the amino acid exchange M1T in *cg3388*	This study
pK19*mobsacB* -dtxR^{T8A}	pK19*mobsacB* with a construct for the amino acid exchange T8A in *dtxR* (cg2103)	This study

pVWEx1	KmR. Ptac. lacI^q *C. glutamicum*/*E. coli* shuttle vector	[86]
pVWEx1-*phe*	Km^R. pVWEx1 overexpressing *phe* with an artificial RBS	This study
pEKEx3	SpecR. Ptac. lacI^q C. glutamicum/E. coli expression shuttle vector	[87]
pEKEx3- *cg2796-cg2797*	Km^R. pEKEx3 overexpressing *cg2796-cg27976* with an artificial RBS	This study
pET16b	Amp^R. Expression plasmid for production of His-tagged proteins	Novagen
pET16b-*cg3388*	Amp^R. pET16b overexpressing *cg3388* with an artificial RBS	This study

Table S3 Growth of *C. glutamicum* WT and C1* with *p*-cresol or glucose as sole carbon source. Cultivation was performed in triplicates in CGXII minimal medium using 500 mL shaking flask contaiing either 2 mM *p*-cresol or the carbon equivalent of glucose (2.3 mM).

Carbon source		Growth of strains	
***p*-Cresol**	**glucose**	**WT**	**C1***
-	2.3 mM	+	+
2 mM	-	+	-
