# Supplementary figures and images for: Physiological Response of Corynebacterium glutamicum to Indole
Source: Microorganisms. 2020 Dec 8;8(12):1945. doi: 10.3390/microorganisms8121945 (PMC7764795; doi:10.3390/microorganisms8121945)

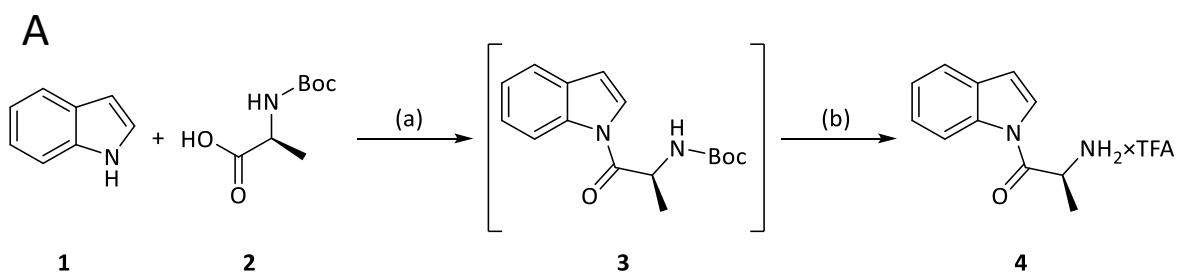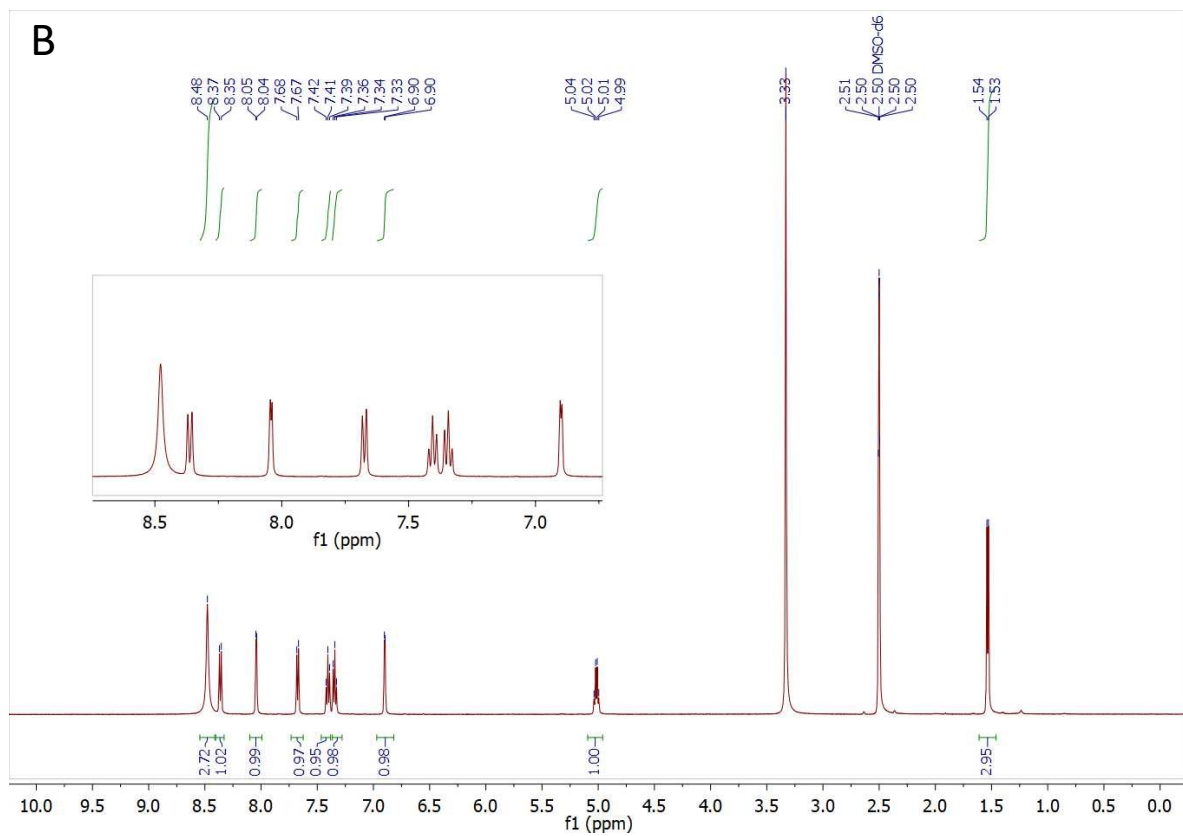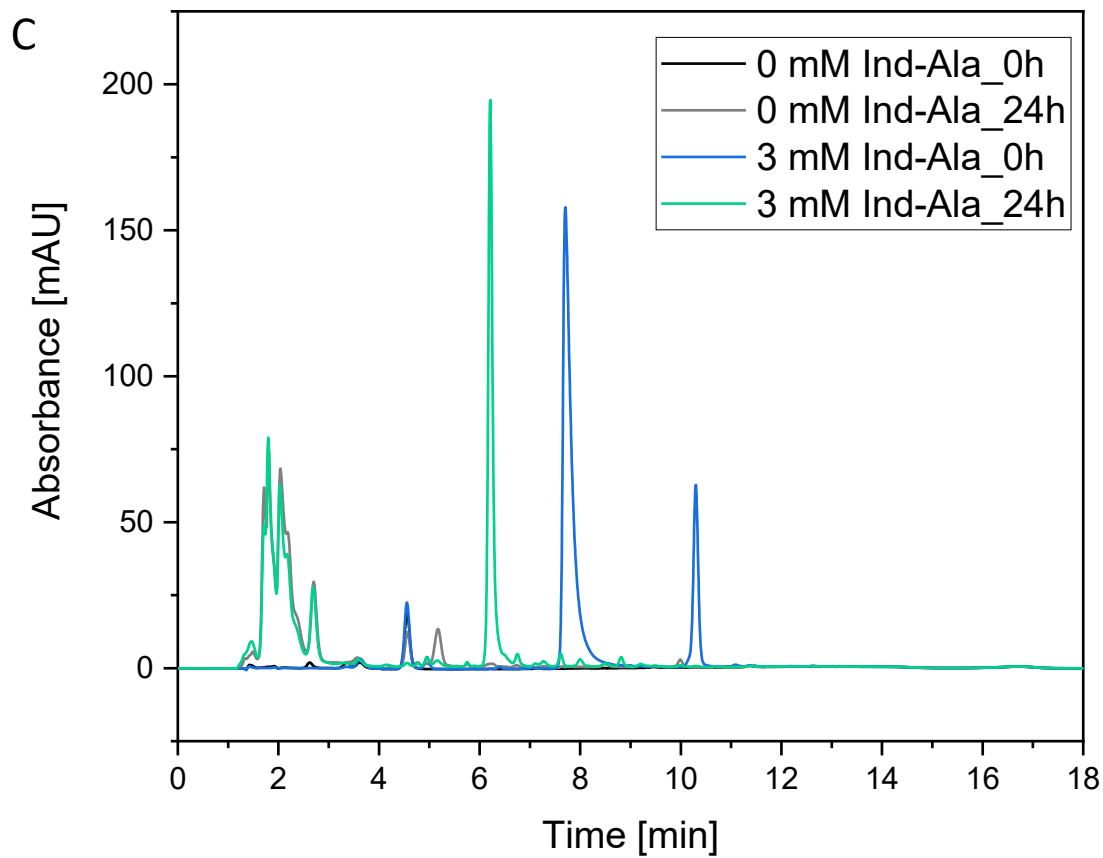

Supplement: Supplementary file 1 [file microorganisms-08-01945-s001.zip › Supplementary Data_indole response/Supplementary Data Figure S1.pdf]

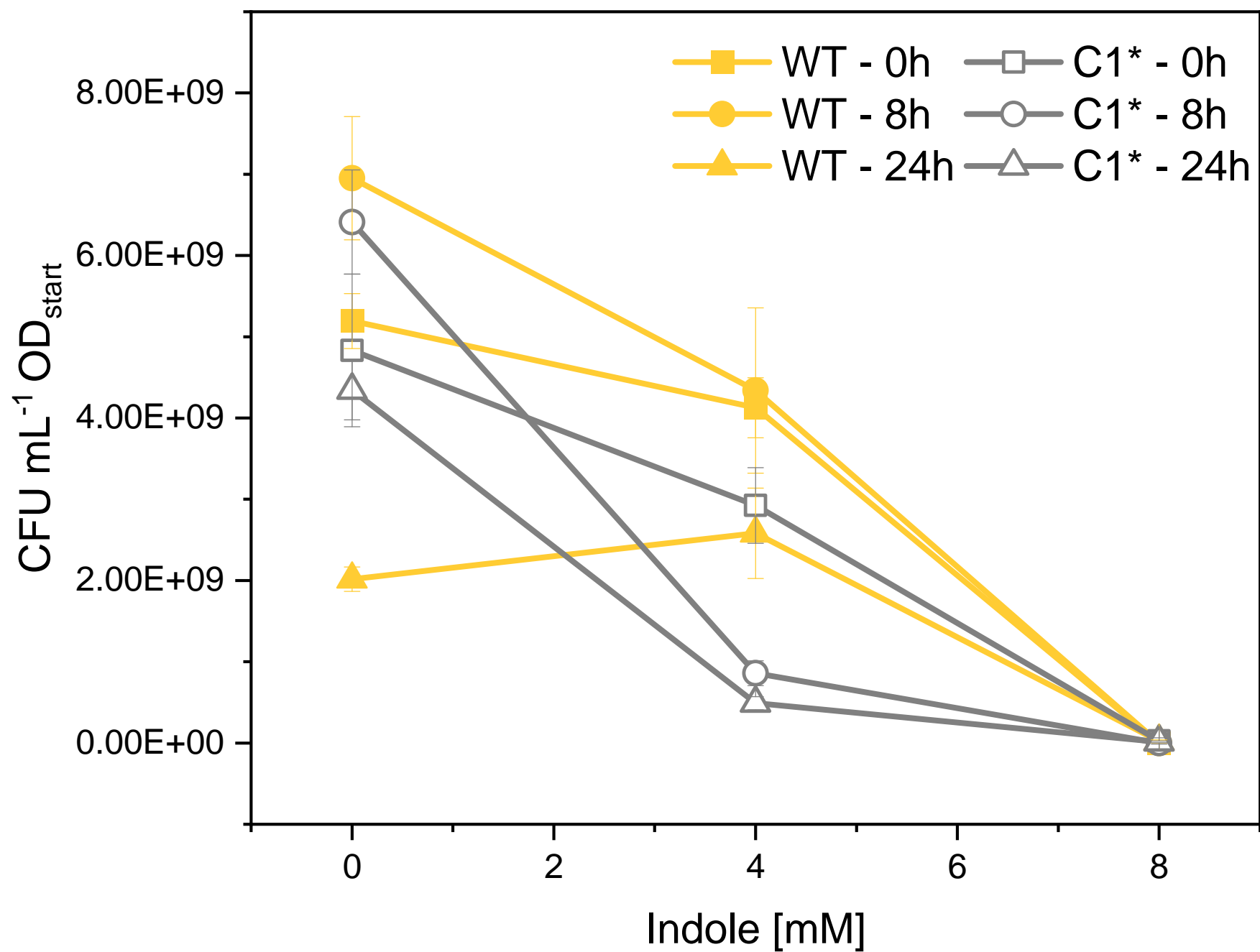

Supplement: Supplementary file 1 [file microorganisms-08-01945-s001.zip › Supplementary Data_indole response/Supplementary Data Figure S2.pdf]

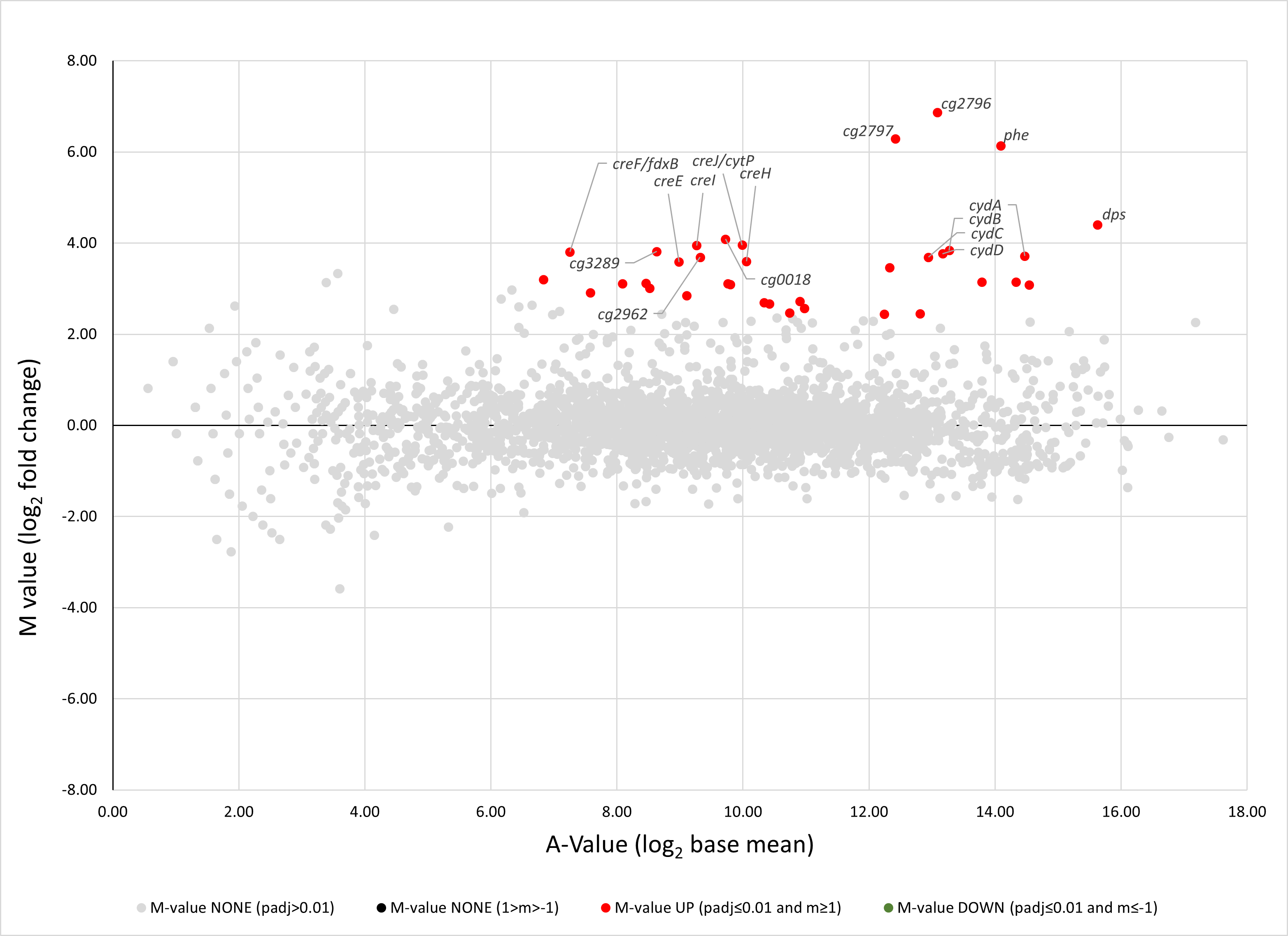

Supplement: Supplementary file 1 [file microorganisms-08-01945-s001.zip › Supplementary Data_indole response/Supplementary Data Figure S3A.png]

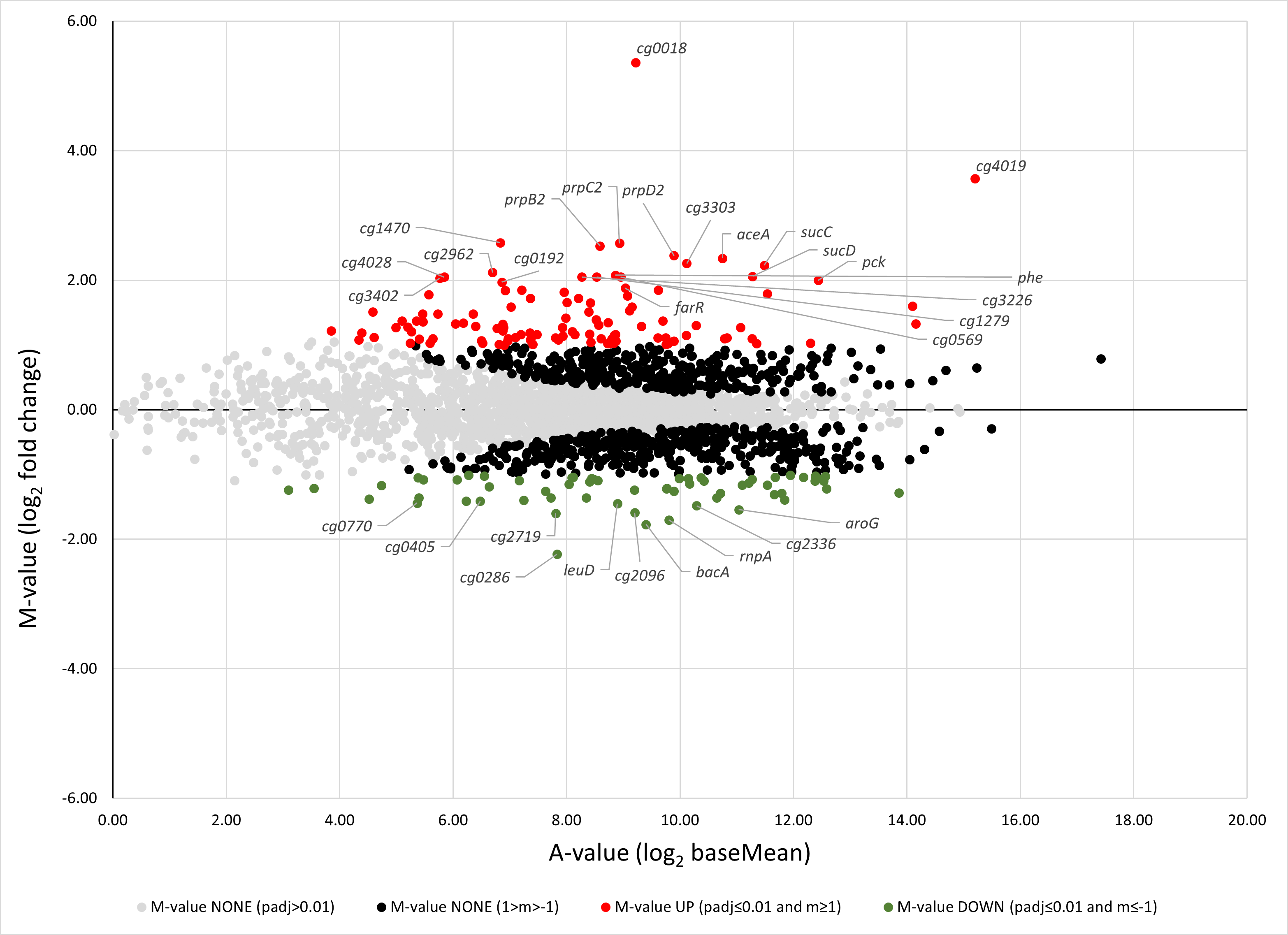

Supplement: Supplementary file 1 [file microorganisms-08-01945-s001.zip › Supplementary Data_indole response/Supplementary Data Figure S3B.png]

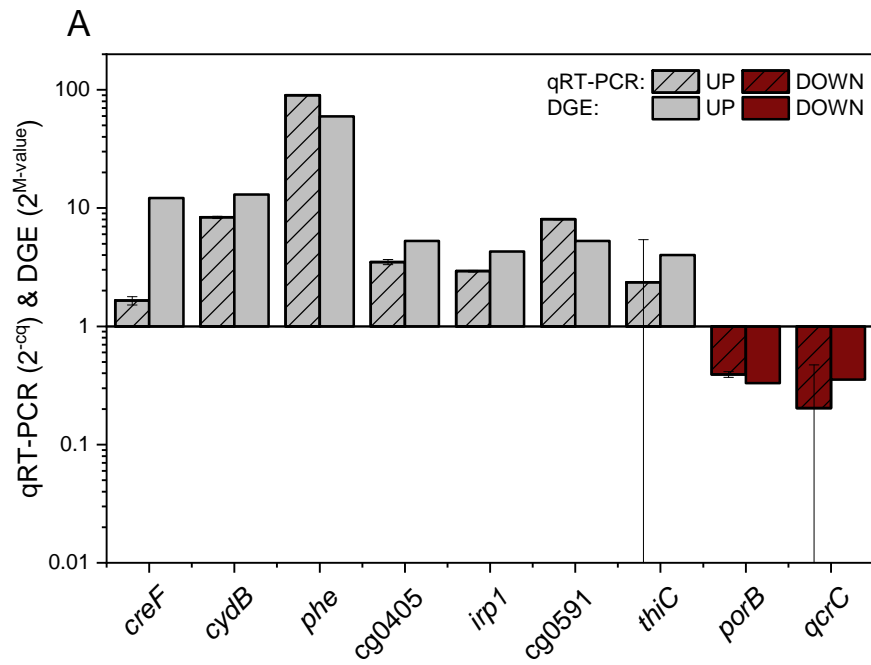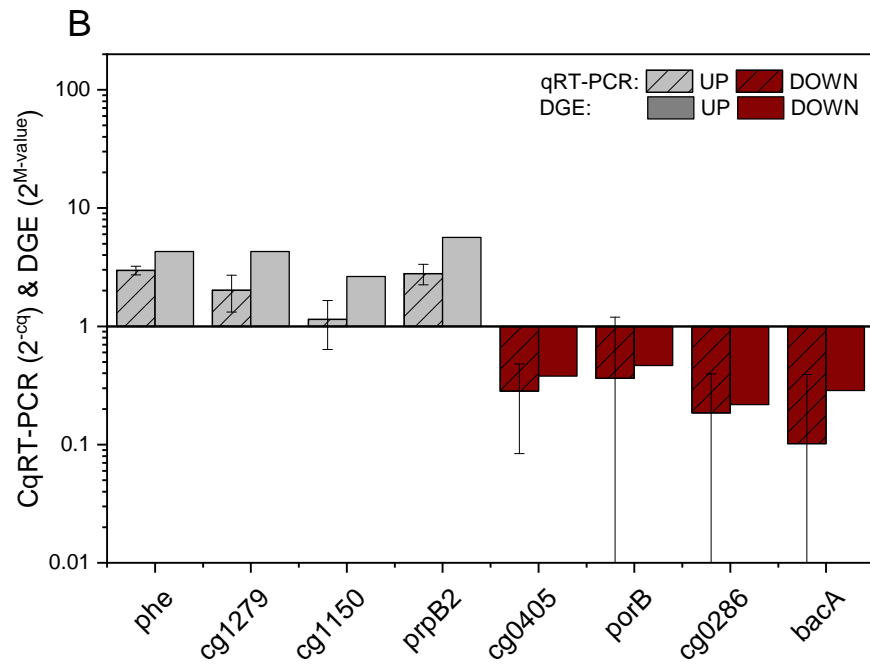

Supplement: Supplementary file 1 [file microorganisms-08-01945-s001.zip › Supplementary Data_indole response/Supplementary Data Figure S4.pdf]

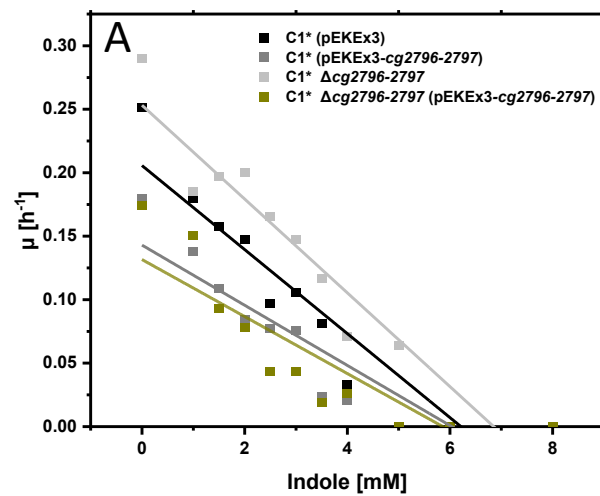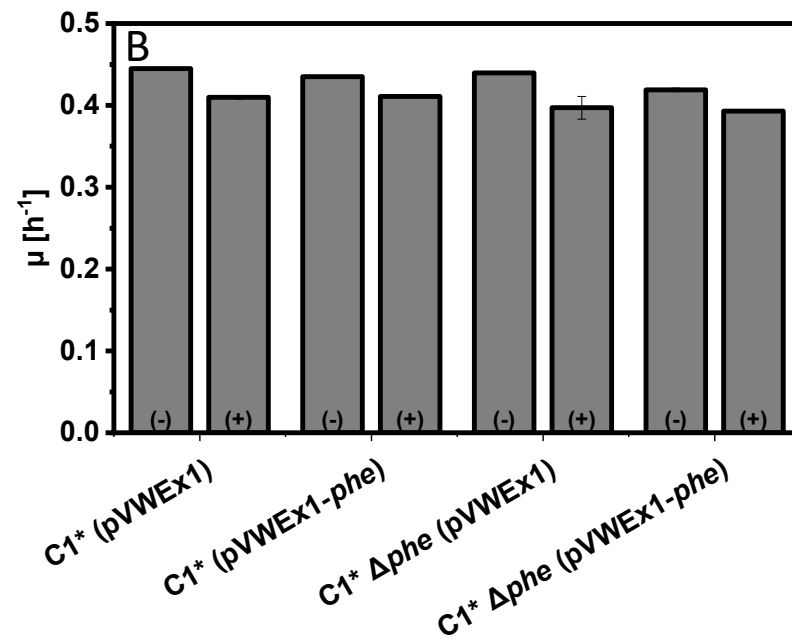

Supplement: Supplementary file 1 [file microorganisms-08-01945-s001.zip › Supplementary Data_indole response/Supplementary Data Figure S5.pdf]

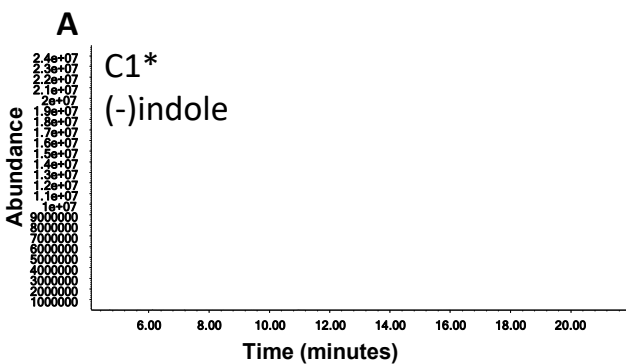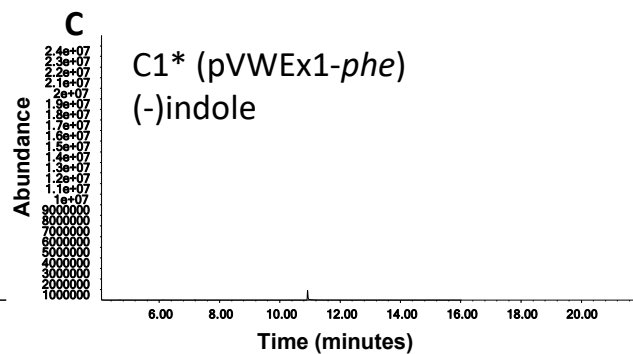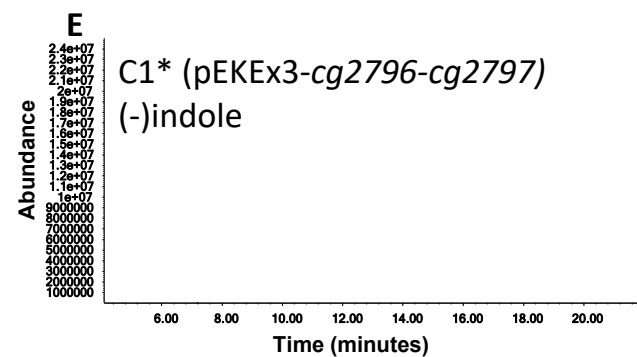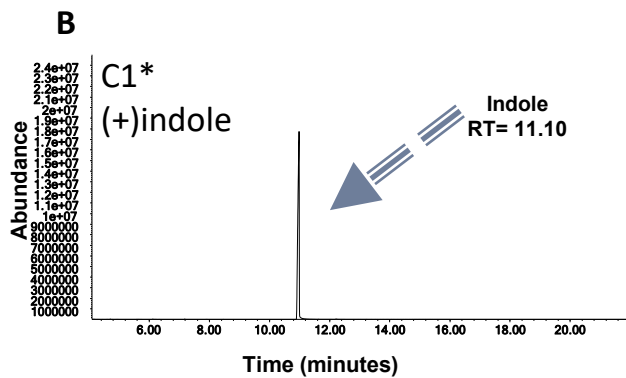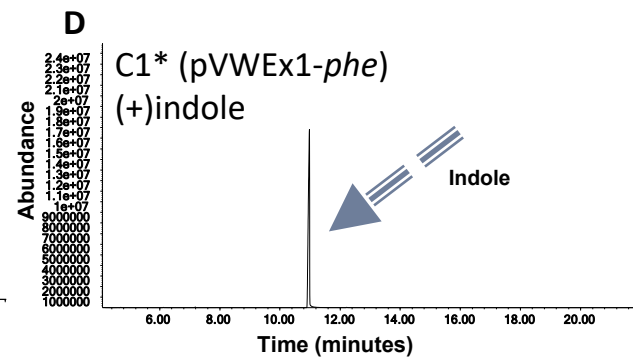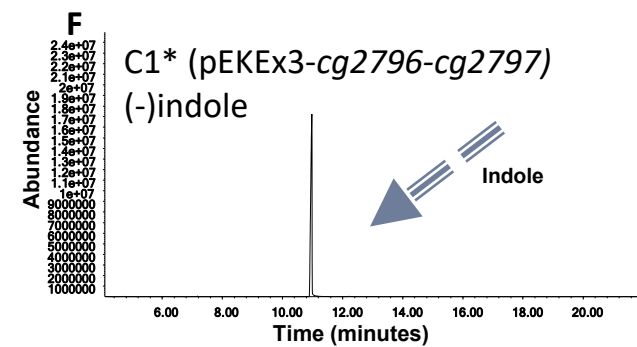

Supplement: Supplementary file 1 [file microorganisms-08-01945-s001.zip › Supplementary Data_indole response/Supplementary Data Figure S6.pdf]

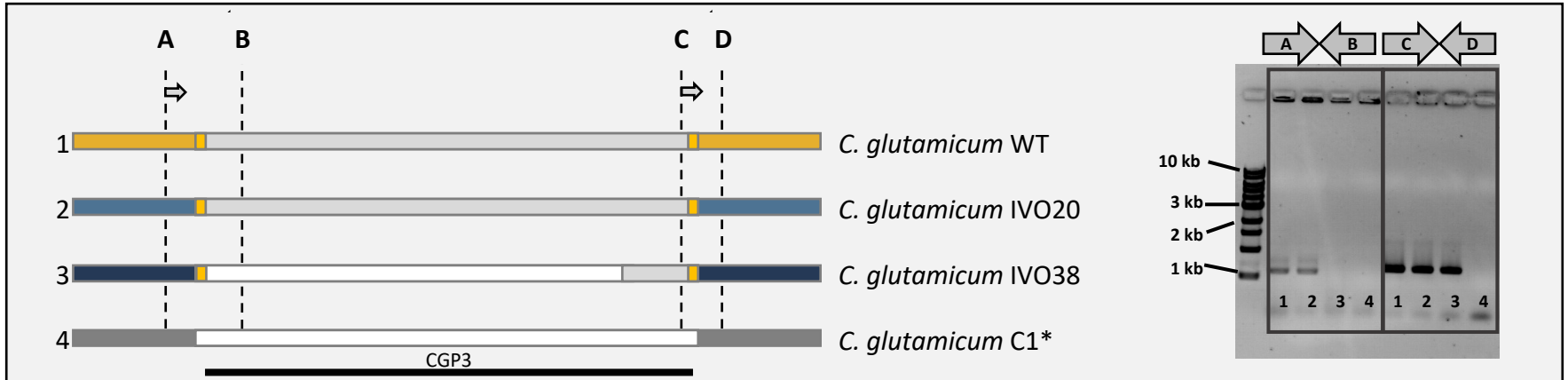

Supplement: Supplementary file 1 [file microorganisms-08-01945-s001.zip › Supplementary Data_indole response/Supplementary Data Figure S7.pdf]
